# Effect of *Lacticaseibacillus rhamnosus* IDCC 3201 on irritable bowel syndrome with constipation: a randomized, double-blind, and placebo-controlled trial

**DOI:** 10.1038/s41598-024-72887-x

**Published:** 2024-09-27

**Authors:** Hyeji Kwon, Eoun Ho Nam, Hayoung Kim, Haneul Jo, Won Yeong Bang, Minjee Lee, Hyeonmin Shin, Dana Kim, Jeongho Kim, Hyejin Kim, Jongkyun Lee, Young Hoon Jung, Jungwoo Yang, Daeyoun David Won, Minhye Shin

**Affiliations:** 1 Immunology Laboratory, Cancer Genomic Research Institute, Seoul Song Do Colorectal Hospital, Seoul, 04597 Republic of Korea; 2 Department of Microbiology, College of Medicine, Inha University, Incheon, 22212 Republic of Korea; 3 Department of Biomedical Science, Program in Biomedical Science and Engineering, Inha University, Incheon, 22212 Republic of Korea; 4 Ildong Bioscience, Pyeongtaek-si, Gyeonggi-do 17957 Republic of Korea; 5 Digestive Endoscopic Center, Seoul Song Do Colorectal Hospital, Seoul, 04597 Republic of Korea; 6 Department of Surgery, Pelvic Floor Center, Seoul Song Do Colorectal Hospital, Seoul, 04597 Republic of Korea; 7 School of Food Science and Biotechnology, Kyungpook National University, Daegu, 41566 Republic of Korea; 8 Institute of Fermentation Biotechnology, Kyungpook National University, Daegu, 41566 Republic of Korea; 9 Department of Microbiology, Dongguk University College of Medicine, 123 Dongdae-ro, Gyeongju, 38066 Republic of Korea

**Keywords:** Gastrointestinal diseases, Metabolomics, Sequencing

## Abstract

Irritable bowel syndrome is a chronic disorder affecting the gastrointestinal tract, negatively impacting patients’ quality of life. Here, we aimed to evaluate the effects of *Lacticaseibacillus rhamnosus* IDCC 3201 (RH 3201) on irritable bowel syndrome with constipation (IBS-C). In this randomised, double-blind, placebo-controlled trial, a total of 30 subjects with IBS-C were randomly assigned (1:1) to receive 8 weeks of probiotics administration or placebo. Concerning bowel activities, both irritant bowel movements and discomfort caused by constipation showed significant improvement with RH 3201 at 8 weeks. Symptoms including severity of abdominal bloating, frequency of abdominal bloating, and satisfaction of bowel habits based on the irritable bowel syndrome-severity scoring system also ameliorated in the probiotic group. Analysis of the fecal microbiome revealed that the abundance of *Bacteroides cellulosilyticus* and *Akkermansia muciniphila* was higher during the period of RH 3201 administration compared to the placebo. Untargeted metabolome analysis further suggested a correlation between specific metabolites, such as N-acetylornithine, xanthine, and 3-phenylpropionic acid, and the improvement of clinical symptoms. These results indicate that RH 3201 was effective in ameliorating IBS-C, potentially by enriching beneficial microbes and associated metabolites in the gut environment.

**Supplementary Information:**

The online version contains supplementary material available at 10.1038/s41598-024-72887-x.

## Introduction

As scientific evidence accumulates regarding the link between dysbiosis of gut microbiota and human diseases, probiotics have garnered significant interest over the last few decades^1^. Recent studies on probiotics have targeted the management of symptoms for various diseases, including Crohn’s disease, depression, obesity, diabetes and cancer. In particular, gut health is increasingly recognized as a crucial factor in combating diseases. This recognition has given rise to concepts such as the gut-organ axis, including the gut-brain, gut-heart, gut-liver and gut-skin axes^2,3^.

The mechanisms of probiotics’ beneficial effects can be summarized as the secretion of functional metabolites, enhancement of gut barrier integrity, immune system boosting, and promotion of water absorption and gut motility^4,5^. Short-chain fatty acids are among the most well-known microbial metabolites, playing a pivotal role in gut and metabolic health^6,7^. Their biological functions encompass maintaining the activity of mucosal immune cells, reducing pH to inhibit pathogenic bacterial growth, and protecting the intestinal epithelial barrier. When dysbiosis occurs due to factors such as antibiotic therapy, intense physical stress, and chronic illness, probiotics can be prescribed to restore balance in the gut microbiome^8^.This leads to a reduction in bacterial translocation, an improvement in tight junction integrity, and stimulation of mucin production^9^. Furthermore, probiotics prevent the penetration and overgrowth of pathogenic microbes in the gut by producing antimicrobial substances and competing for space and nutrients^10^. Notably, probiotics may increase water absorption and gut motility, thereby preventing diarrhea and constipation^11,12^. Pathogenic bacteria often disrupt aquaporins, water-channel membrane proteins, which can increase water content in stool and lead to colon dehydration^12^.

Irritable bowel syndrome (IBS) is a type of disorder of gut-brain interactions (DGBIs), previously known as functional gastrointestinal disorders, characterized by recurrent abdominal pain associated with defecation or alterations in stool frequency and form^13^. Bloating is also a commonly accompanying symptom. The symptoms of IBS significantly affect quality of life, work productivity, and healthcare costs, highlighting the need for effective treatment and management strategies. While the pathogenesis of IBS remains elusive, an increasing array of therapeutic agents, including pharmacotherapy, lifestyle modifications, and dietary manipulation, have been suggested for its treatment^14^. Multiple studies have demonstrated that the administration of probiotics can alleviate IBS and its symptoms. These studies include both the single use of specific strains such as *Bacillus coagulans* LBSC^15^ or *Bifidobacterium lactis* DN-173 010^16^, as well as combinations of several species, predominantly *bifidobacteria* and *lactobacilli*^17–19^. Potential mechanisms for the action of probiotics in IBS have been proposed, including inhibition of pathogen binding, enhancement of barrier function, anti-inflammatory effects, alterations in visceral hypersensitivity, and modification of gut microbiota^20^.

Previously, we demonstrated that tyndallized *Lacticaseibacillus rhamnosus* IDCC 3201 (RH 3201) ameliorates atopic dermatitis through the regulation of hypersensitive immune reactions^21^. Our previous research also established the anti-inflammatory and anti-pathogenic effects of RH 3201 in the context of gut health^22^. Moreover, in a high-protein diet mouse model, RH 3201 was shown to enhance protein digestibility and elevate plasma concentrations of amino acids^4^. Building upon these findings, the current study was designed as a randomized, double-blind, placebo-controlled trial to assess whether RH 3201 can improve the symptoms of IBS with constipation (IBS-C). To gain insights into the mechanisms underlying gut health, we conducted comprehensive analyses of microbiota and metabolites in fecal samples. This investigation contributes valuable knowledge regarding the pivotal role of probiotics in modulating gut microbiota and promoting gut health.

## Methods

### Ethics information

This study was approved by the clinical research ethics committee of Seoul Songdo Hospital (approval number: 2022-003) and registered at the CRIS (Clinical Research Information Service) under registration number KCT0009026 (Date of first trial registration on 08/12/2023). All experimental protocols were in accordance with the Declaration of Helsinki (2013). Prior to enrollment, all participants were provided with the information concerning the purpose, expected effects, unexpected side effects, and study method of the study and voluntarily provided written consent.

### Subjects and study design

This study was conducted at Seoul Songdo Hospital from June 1st, 2022 to March 31st, 2023. Participants qualified for randomization had clinical responses based on the Rome IV criteria: (1) experiencing hard or lumpy stools at least 1 out of 4 bowel movements or having loose stools in fewer than 1 out of 4 bowel movements; (2) experiencing abdominal bloating, abdominal pain; and (3) a sensation of incomplete evacuation after bowel movements^23^. The exclusion criteria were as follows: (1) individuals diagnosed with constipation other than constipation-predominant irritable bowel syndrome; (2) individuals with a history of gastrointestinal surgery, except for appendectomy and cesarean section; (3) individuals with warning signs including rectal bleeding and unexplained weight loss; (4) individuals with gastrointestinal conditions (e.g., colitis, colorectal cancer, and other structural conditions) currently or within the past 2 years that could affect the assessment of gastrointestinal function; (5) individuals who have taken medications within the past 2 weeks that could affect gastrointestinal motility, constipation and diarrhea; (6) individuals with concurrent disorders including liver cancer, cirrhosis, chronic kidney disease, and congestive heart failure; (7) (expected) pregnant or breastfeeding women; (8) individuals with psychiatric disorders including depression and schizophrenia; (9) individuals with hypersensitivity to the investigational product or its components or allergies to specific ingredients. The composition of investigational products (IPs) are shown in Supplementary Table S1 and IPs were identical in shape and color between placebo and RH 3201. It is noted that an orange color powder was added only to the placebo to match its color with RH 3201, which was slightly orange-colored due to the manufacturing process. Freeze-dried viable RH 3201, provided by Pharmcross (Hwaseong, Gyeonggi-do, Korea), was orally administered at a dosage of 1 × 10^10^ CFU/capsule.

**Fig. 1:**
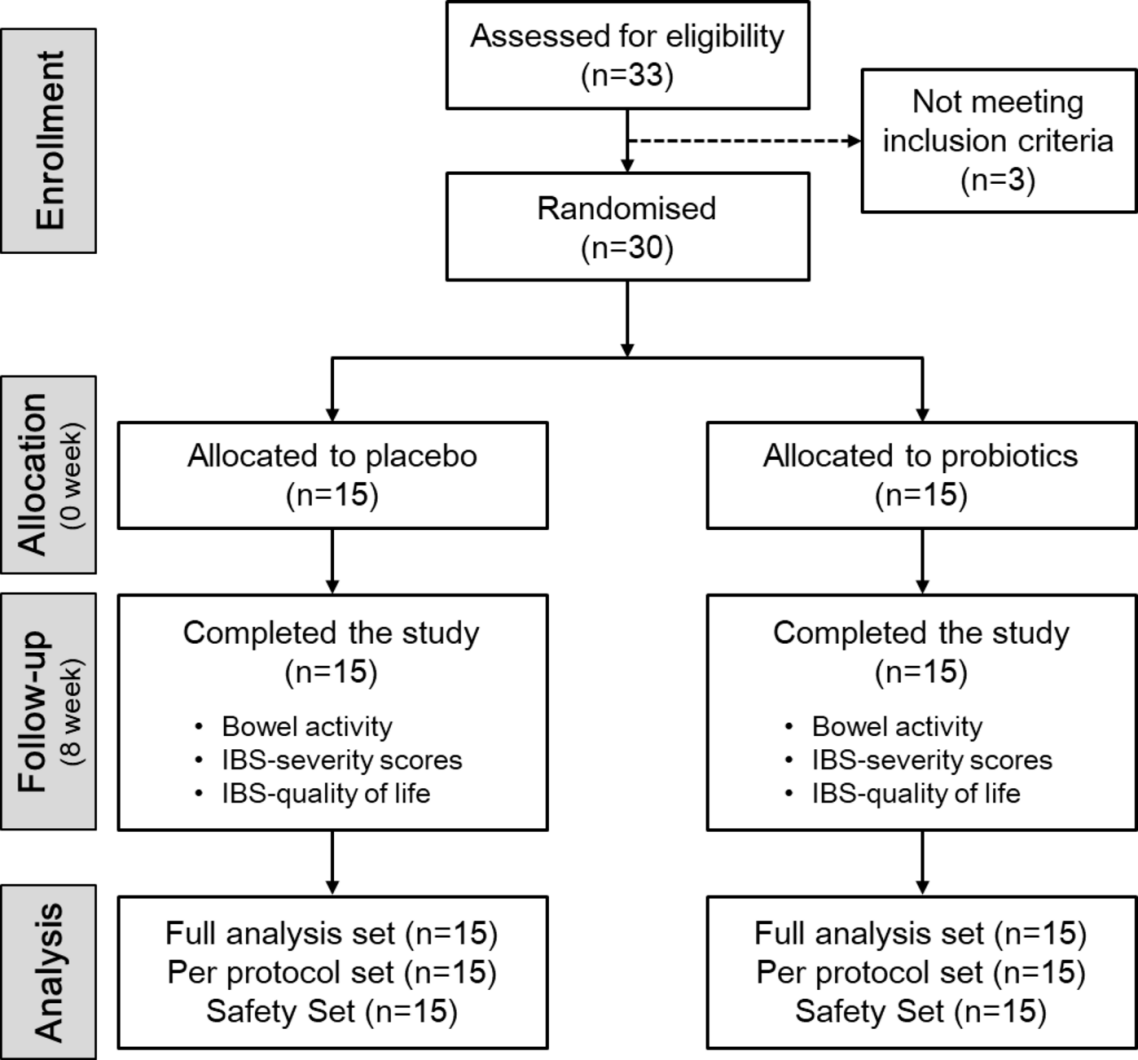
CONSORT flow diagram in this study.

This study was an 8-week randomized, double-blind, placebo-controlled clinical trial, consisting of two phases; a 2-week baseline run-in observation period (-2 to 0 week) and an 8-week intervention period (Fig. 1 and Supplementary Table S2). All participants were enrolled based on the inclusion and exclusion criteria. Sample size was calculated based on a previous clinical trials on IBS-C patients showing differences more than 50% between the two groups were considered clinically significant^24,25^. The probability of a type I error was set at 0.05, and statistical power was 80%. Based on these conditions, 15 participants in each group were enrolled. The research sponsor divided the patients into two groups of 15 adults using a computer-generated random list and randomly assigned codes matching the manufactured products. These codes were then delivered to the researchers. The researchers remained blinded when supplying the selected test products corresponding to the randomly assigned codes to the study participants. Post-entry visits were conducted at the end of weeks 4 and 8. Based on the remaining proportion of returned investigational products at the 4-week and 8-week visits, compliance was determined as low if it fell below 80% for two consecutive assessments. If it exceeded 100%, it was recorded as such but capped at 100%. Compliance was calculated as the ratio of actual consumption to the prescribed amount, multiplied by 100. The full analysis, per-protocol, and safety set were defined as 30 participants who completed the clinical test, because all the participants were not excluded during the study (Fig. 1).

### Data collection and analysis of questionnaires

Participants completed questionnaires of bowel habits, IBS-SSS (IBS-Severity Scoring System), and a quality of life (QOL) at 0, 4, and 8 weeks^26^. The questionnaires for bowel habits were designed to measure 12 characteristics, including number of bowel movement per week, defecation time, amount of feces, number of times of irritant bowel movements, number of times when bowel movements felt incomplete, shape of the feces, number of times of abdominal pains before bowel movements, number of times of abdominal pains during bowel movements, degree of abdominal, amount of gas, discomfort after bowel movement, and discomfort due to constipation^27^. The IBS-SSS (IBS-Severity Scoring System) questionnaire is a tool that assesses the severity of abdominal pain, frequency of abdominal pain (number of days with abdominal pain in the past 10 days), severity of abdominal distension, dissatisfaction with bowel habits, and interference with daily activities. Each of these was scored on a scale of 0 to 100 and when summed, they resulted in a total score ranging from 0 to 500. The severity of IBS-C is defined within the following ranges: mild 75–174, moderate 175–300, and severe > 300^28^. The IBS-QOL (IBS Quality of Life) questionnaire consists of 34 specially designed items to assess quality of life impairment due to IBS-C symptoms^29^. Each item is scored on a 5-point scale representing one of eight dimensions (dysphoria, interference with activity, body image, health worry, food avoidance, social reactions, sexual, and relationships). To derive the overall score for IBS-related QOL, scored items were summed and converted to a scale of 0 to 100 for ease of interpretation, where 0 represents poor quality of life, and 100 represents the highest quality of life. Finally, we evaluated the differences between the two groups using non-parametric statistical methods such as the Mann-Whitney U test or the Wilcoxon signed-rank test. Differences among time points (0, 4, 8 weeks) were analyzed using repeated measures ANOVA with Tukey’s post-hoc analysis. All analyses were conducted using Jamovi software. A significance level of *p* < 0.05 was considered statistically significant.

### Fecal sample collection

Two samples per visit were collected from each participant at 0, 4, and 8 weeks. The fecal sample collection kit (AccuStool DNA Preparation Kit, AccuGene, Incheon, Korea) consists of two types of tubes, one containing buffer solution for 16 S metagenome analysis and the other without the solution for metabolome analysis. The study participants were provided with the AccuStool kit to collect fecal samples by themselves at week 0, 4 and 8. It was distributed in advance during the visits at -2 weeks, 0 week, and 4 weeks. The fecal samples were collected at the participants’ homes 2–3 days prior to the visits, stored at -20 ℃, and then submitted during the visits. At the visit sites, fecal samples were stored at -80℃ until microbiota and metabolite analysis. It is noted that stool samples were collected in special tubes containing a preservation buffer, which ensures sample stability for up to three months during storage and transportation. This stability has been validated by the manufacturer, ensuring the accuracy of microbial DNA in subsequent analyses.

### DNA extraction

Approximately 200 mg of each fecal sample was extracted using the commercial QIAamp Power Fecal Pro DNA kit (Qiagen, Hilden, Germany). The concentration and purity of DNA were measured using a UV spectrophotometer (Eppendorf, Hamburg, Germany). The concentration of double-stranded DNA was quantified using the Qubit dsDNA HS Analysis Kit (Invitrogen, Eugene, OR, USA).

### PCR analysis of RH 3201

The PCR was performed using Q5^®^ High-Fidelity 2x Master Mix (New England Biolabs, Ipswich, MA). The primer sequences are as follows: Forward, TGCTTGCATCTTGATTTAATTTTG; and Reverse, GGTTCTTGGATYTATGCGGTATTAG. PCR reactions were conducted under the following conditions: activation for 5 min at 95 °C, initial denaturation for 15 s at 95 °C, annealing for 15 s at 62 °C, and extension for 30 s at 72 °C. A total of 25 cycles were performed from denaturation to extension. Finally, all products after the cycles hold for 5 min at 72 °C. Subsequently, the products were analyzed by 1% (w/v) agarose gel electrophoresis with a 1 kb DNA ladder (Enzynomics, Daejeon, Korea), and the product bands for 122 bp were visualized using a GelDoc imaging system (Bio-Rad, Hercules, CA).

### Sequencing library preparation and 16 S rDNA sequencing

Sequence libraries were prepared to amplify V3–V4 regions of the 16 S rRNA gene using 16 S Microbiome NGS Assay Library Preparation Kit (ViennaLab Diagnostics GmbH, Vienna, Austria). The V3-V4 regions of bacterial 16 S rRNA genes were amplified using the PCR1 V3-V4 Primer Mix (ViennaLab Diagnostics GmbH). The reaction conditions consisted of an initial denaturation at 95 °C for 3 min, followed by 20 cycles of denaturation at 95 °C for 15 s, annealing at 55 °C for 15 s, extension at 72 °C for 30 s, and a final elongation at 72 °C for 10 min. The PCR products were purified using Magnetic Beads (ViennaLab Diagnostics GmbH). After purification, the purified eluate was subjected to a second PCR amplification using PCR2 indexing Primers (ViennaLab Diagnostics GmbH) to construct the final libraries with indexes. The cycling conditions for the PCR were the same as the first PCR, except for 10 cycles. The PCR products were further purified using Magnetic Beads (ViennaLab Diagnostics GmbH). To verify the size distribution of PCR amplicons, the template size distribution was assessed using TapeStation D1000 screen tape (Agilent Technologies, Waldbronn, Germany). The pooled libraries were sequenced using an Illumina MiSeq instrument with the MiSeq^®^ Reagent kit v2 (500 cycles) (Illumina, San Diego, CA, USA).

### Statistical analyses for metagenome data

The paired-end MiSeq Illumina reads (2 × 250 bp) data were processed using QIIME 2 version 2022.2^30^ and the ViennaLab Intestinal Microbiome Analysis webtool (ViennaLab Diagnostics GmbH). The Chao1, Shannon, and Simpson indices were computed within QIIME 2 platform to assess α-diversity. Significant differences between the placebo and RH 3201 groups were determined using the Shapiro-Wilk test for normal distribution, Wilcoxon rank-sum test for non-parametric data, and an independent sample t-test for parametric data. Beta diversity was analyzed by performing Non-metric multidimensional scaling (NMDS) plot using Bray-Curtis dissimilarity distances between the groups. To assess significant differences, PERMANOVA analysis performed using the “adonis2” function. Finally, data were visualized using R software ver. 4.2.0 (R Foundation for Statistical Computing, Vienna, Austria) and the ggplot2 and ggpubr graphics packages for R^31,32^. For composition analysis of microbiota between the groups, the relative abundance was calculated using the ViennaLab Intestinal Microbiome Analysis webtool. Taxonomic groups were clustered at the levels of phylum, genus, and species. An independent sample t-test was employed to determine statistically significant differences in the relative abundance. Furthermore, linear discriminant analysis effect size (LEfSe) analysis was performed to identify species showing significant differences between groups^33^.

### Fecal metabolome extraction

Metabolites of fecal samples were extracted for global metabolome analysis. For each 20 mg of sample, 100 µL of deionized water and 500 µL of methanol were added and vortexed vigorously for 10 min to homogenize the samples. After centrifugation at 13,000 rpm for 5 min at 4 °C, the supernatant was collected and filtered through a 0.2 μm PVDF syringe filter. Four hundred microliter of each sample was transferred into a 1.7 ml micro-tube for drying, and the remaining samples were stored in a -80 °C deep freezer. The filtered sample was dried by a vacuum concentrator (VS-802, Visonbionex, Gyeonggi-do, Korea).

### GC-MS analysis

The dried samples were derivatized by adding 30 µL of a solution of 20 mg/mL methoxyamine hydrochloride in pyridine (Sigma, St. Louis, MO, USA) and incubated for 90 min at 30 °C. Sequentially, 50 µL of N, O-bis(trimethylsilyl)trifluoroacetamide (BSTFA; Sigma) was added and heated for 30 min at 60 °C. A mixture of alkane standards and fluoranthene was used as retention indices and an internal standard, respectively. GC-MS analysis was conducted using a Thermo Trace 1310 GC (Thermo, Waltham, MA, USA) coupled to a Thermo ISQ LT single quadrupole mass spectrometer (Thermo), using a DB-5MS column (60-m length, 0.25 mm i.d., and 0.25-µm film thickness) (Agilent, Santa Clara, CA, USA). Derivatized samples were injected at 300 °C using a split ratio of 1:5, and metabolites were separated using a helium flow of 1.5 mL/min using the following oven program; 2 min at 50 °C, 50 °C to 180 °C at 5 °C/min, 8 min at 180 °C, 180 °C to 210 °C at 2.5 °C/min, 210 °C to 325 °C at 5 °C/min, and 10 min at 325 °C. Mass spectra were acquired in the scan range 35–650 m/z at 5 spectra per second in electron impact ionization mode and an ion source temperature of 275 °C. Spectra were processed using Thermo Xcalibur and AMDIS softwares with automated peak detection, and metabolites were identified by matching mass spectra and retention indices using the NIST Mass spectral search program (version 2.0, Gaithersburg, MD, USA) and MS-DIAL (http://prime.psc.riken.jp/compms/msdial/main.html). Relative metabolite intensities were normalized by the sum of identified peaks.

### Statistical analyses for metabolome data

Metabolome data were evaluated by paired t-test and One-way ANOVA using GraphPad Prism 9 (San Diego, CA, USA). Differences were considered significant when p or P values < 0.05 (*) or < 0.01 (**). Multivariate analyses and heatmap generation were performed using MetaboAnalyst 5.0 (https://www.metaboanalyst.ca/). Spearman’s correlations among the clinical data, microbiota, and metabolites were calculated and visualized using Microsoft Excel (Microsoft, Redmond, WA, USA) and GraphPad Prism 9.4.1 (San Diego, CA, USA).

## Results

### *L. rhamnosus* IDCC 3201 (RH 3201) improves symptoms of irritable bowel syndrome with functional constipation (IBS-C)

In order to assess whether RH 3201 can improve the symptoms of IBS-C, we conducted a randomized, double-blind, and placebo-controlled trial of RH 3201 in IBS-C patients and evaluated clinical outcomes based on clinical response described in Methods. The baseline characteristics of the study participants are presented in Supplementary Table S3. The number of participants who completed the entire study protocol in the placebo and RH 3201 groups were 15 each, with ages of 37.1 ± 13.7 and 36.4 ± 13.6, respectively. The window period was set at ± 7 days, and all participants completed the intervention within this period. No participants were disqualified due to low compliance, and the average compliance rate was 96%. The presence of the administered RH 3201 was confirmed by PCR and supporting relative abundance of the genus (Supplementary Fig. S1). It is noted that *L. rhamnosus* is commonly found in healthy individuals, particularly in the female genitourinary tract, and it is often used in dairy products or supplemented as probiotics. Consequently, there is a possibility of detecting *L. rhamnosus* in the placebo group, which could introduce confounding factors. No significant differences were observed between the groups in terms of basic characteristics such as body size, physical activity, blood pressure, and health habits related to constipation such as alcohol consumption and smoking. Furthermore, this difference was not observed after the 8-week prescription either (Supplementary Table S3 and S4).

**Fig. 2:**
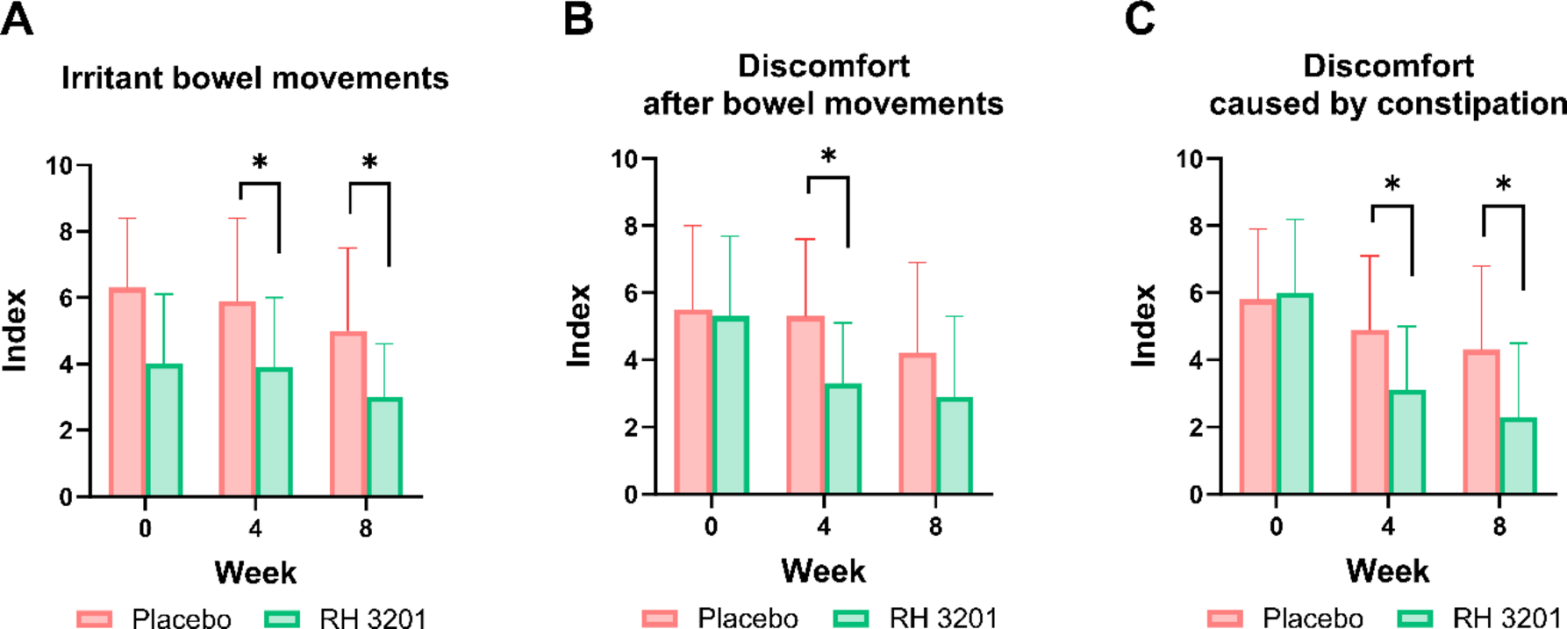
Assessment of bowel activities in participants by RH 3201. (**A**) Number of times of irritant bowel movements. (**B**) Discomfort after bowel movements. (**C**) Discomfort caused by constipation. Data were expressed as mean ± standard deviation. Significant differences compared to the placebo group are indicated as * (*p* < 0.05) using independent samples t-test.

**Fig. 3:**
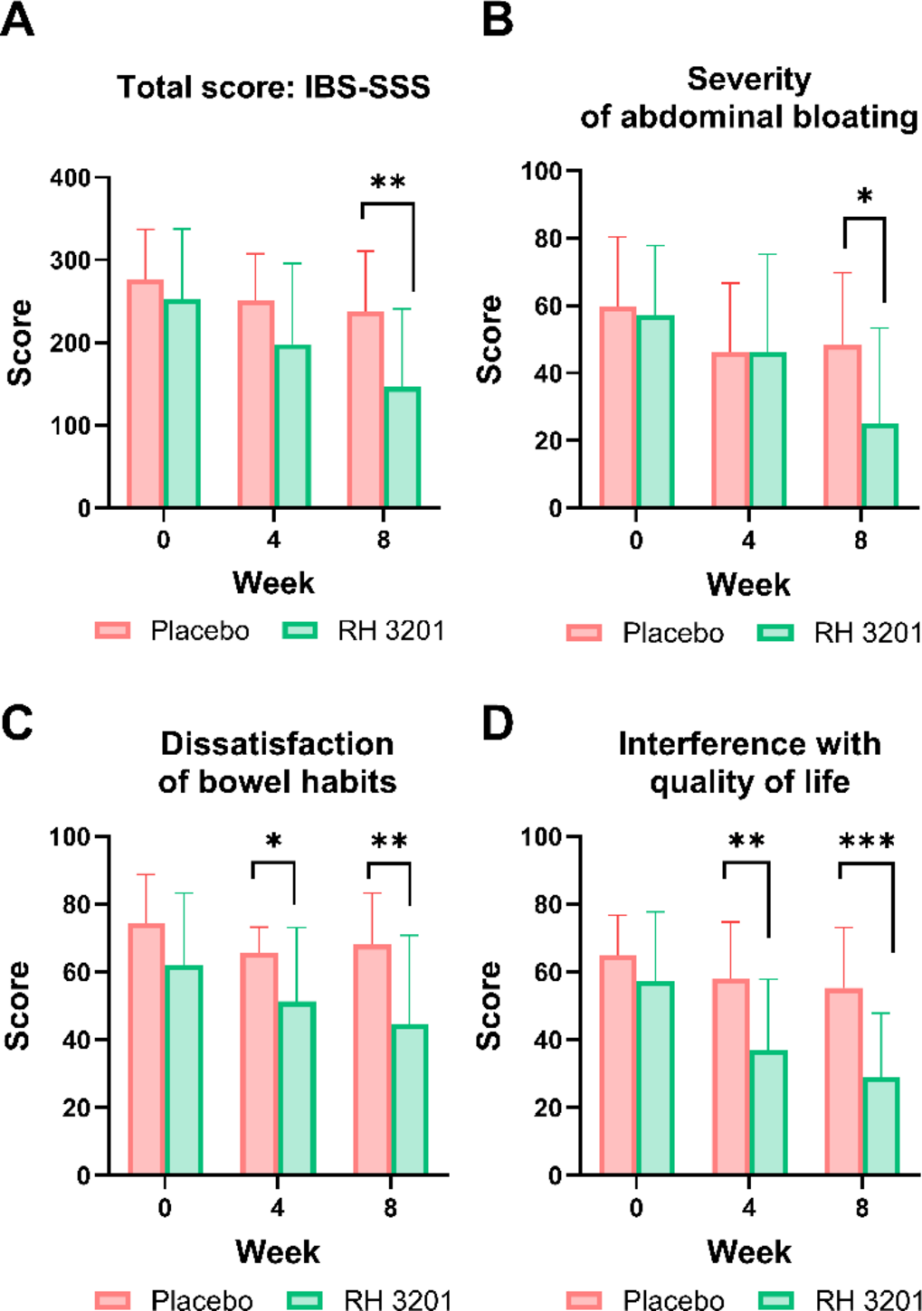
Assessment of IBS-SSS scores in participants by RH 3201. (**A**) Total IBS-SSS score. (**B**) Severity of abdominal bloating. (**C**) Dissatisfaction of bowel habits. (**D**) Interference with quality of life. Data were expressed as mean ± standard deviation. Significant differences compared to the placebo group are indicated as * (*p* < 0.05), ** (*p* < 0.01) and *** (*p* < 0.001) using independent samples t-test.

**Fig. 4:**
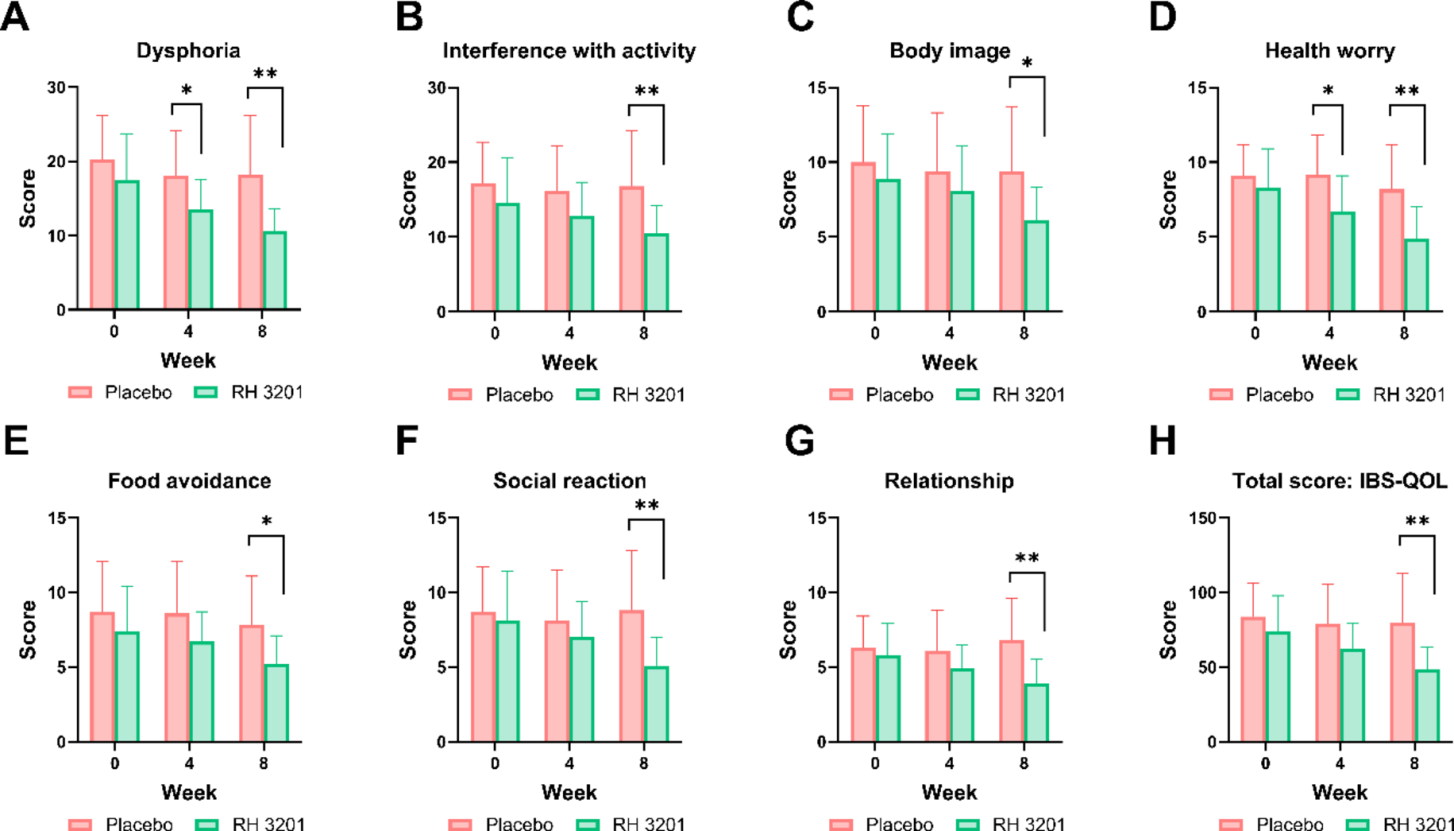
Assessment of IBS-QOL (Quality Of Life) in participants by RH 3201. (**A**) Dysphoria. (**B**) Interference with activity. (**C**) Body image. (**D**) Health worry. (**E**) Food avoidance. (**F**) Social reaction. (**G**) Relationship. (**H**) Total QOL score. Data were expressed as mean ± standard deviation. Significant differences compared to the placebo group are indicated as * (*p* < 0.05) and ** (*p* < 0.01) using independent samples t-test.

Next, we investigated whether RH 3201 would improve gut health using a 12 bowel habits questionnaire, including the number of bowel movements per week, defecation time, and the amount of feces (Fig. 2 and Supplementary Table S5). In the results, the frequency of irritable bowel movements and discomfort caused by constipation significantly improved for 8 weeks of RH 3201 prescription at a dose of 1 × 10^10^ CFU (Fig. 2A and C), whereas discomfort after bowel movements was significantly ameliorated only at 4 weeks (Fig. 2B). In the IBS-SSS (severity scoring system) scores, overall scores showed significant improvement of the IBS-C symptoms at 8 weeks (Fig. 3A and Supplementary Table S6) (*p* = 0.006). More specifically, 3 out of 5 items in IBS-SSS scores significantly improved either at 4-week or 8-week: severity of abdominal bloating, dissatisfaction of bowel habits, and interference with daily activities (Fig. 3B-D). Furthermore, the total score of IBS-QOL (quality of life) was significantly lower in the RH 3201 group than in the placebo group at 8 weeks (*p* = 0.003) (Fig. 4 and Supplementary Table S7). The items included dysphoria, interference with activity, body image, health worry, food avoidance, social reaction, and relationship. Similar to the results of the comparison between RH 3201 and placebo, RH 3201 significantly improved the indices over the administration period, while no longitudinal changes in clinical symptoms were found in the placebo group (Supplementary Fig. S2-4).

The percentage of responders was evaluated based on the FDA IBS-C responder endpoint^34^. The FDA responder endpoint criteria for IBS-C were as follows: (1) an improvement of at least 30% in their daily worst abdominal pain; (2) an increase of 1 or more complete spontaneous bowel movement per week from baseline; and/or (3) improvement in abdominal pain and complete spontaneous bowel movement during the same week for at least 50% of the weeks of treatment. By applying the criteria, we determined that 73.3% (11/15) of patients achieved the FDA response endpoint. Specifically, 53.3% (8/15) and 66.7% (10/15) of patients taking RH 3201 reported improvements in abdominal pain and bowel movement, respectively, compared to 13.3% (2/15) and 40.0% (6/15) of patients taking placebo, respectively. Thus, it was concluded that RH 3201 improved symptoms of IBS-C, based on the analysis of bowel habits, IBS-SSS scores, and IBS-QOL.

### Administration of RH 3201 is associated with changes in fecal microbiota

**Fig. 5:**
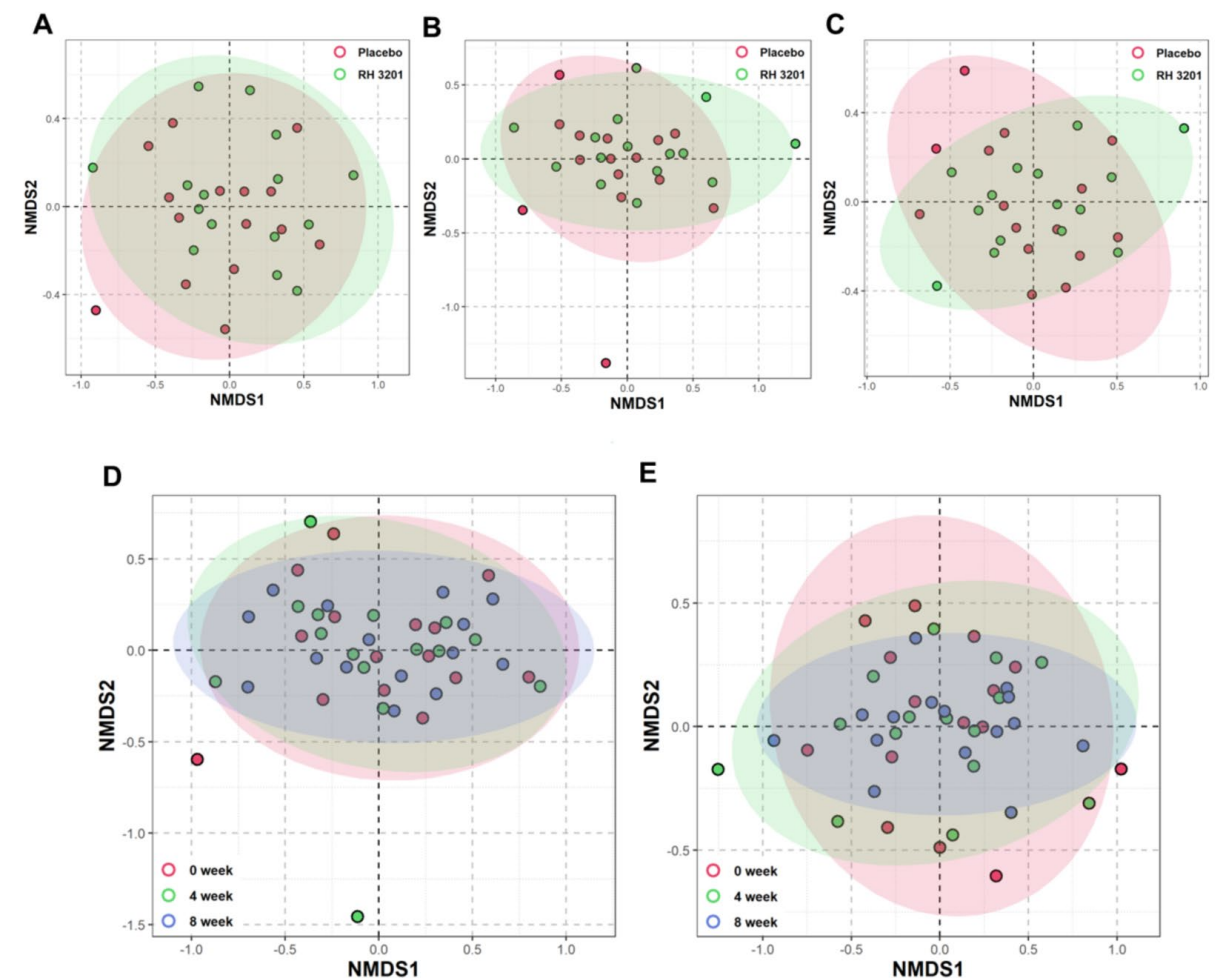
Beta-diversity using non-metric multidimensional scaling (NMDS) and the Bray-Curtis dissimilarity index of fecal microbiome from the placebo and RH 3201 groups. Microbial communities of (**A**) baseline (0-week), (**B**) 4-week, and (**C**) 8-week between the placebo and RH 3202 groups. (**D** and **E**) Microbial communities in (**D**) placebo and (**E**) RH 3201 group during the intervention periods.

To elucidate the causal effects of RH 3201 administration on the improved clinical symptoms in bowel habits and IBS-SSS, we analyzed the metagenome profiles of the fecal microbiota. A total of 90 fecal samples were examined between the placebo and RH 3201 groups at 0, 4, and 8 weeks of intervention. A total of 2,386,155 reads were obtained from sequencing, with an average of 26,513 ± 4,886 reads per sample. Alpha-diversity was estimated using Chao1, Shannon, and Simpson indices, commonly employed to quantify the richness, evenness, and total number of microbial species within a single sample (Supplementary Fig. S5). Significant differences were observed only with the Simpson index at 4 weeks, indicating an increase in the diversity of the RH 3201 group (Supplementary Fig. S5C). Regarding β-diversity analysis using Nonmetric Multidimensional Scaling (NMDS), slight variations were seen between the placebo and RH 3202 groups during the intervention period (Fig. 5A-C). In the placebo group, there was no significant change between intervention and baseline microbiota (Fig. 5D), whereas in the RH 3201 group, a microbial shift was observed in the intervention microbiota compared to the baseline microbiota (Fig. 5E).

From the 90 fecal samples, 19 phyla, 328 genera, and 553 species were identified. At the phylum level, Bacillota (63.6%), Bacteroidota (24.7%), Actinomycetota (7.1%), and Pseudomonadota (2.9%) were determined as the four most predominant bacterial phyla in the fecal microbiota (Supplementary Fig. S6A). Collectively, these four dominant phyla contributed to over 98% of the average abundance. At the genus level, the top 22 genera with relative abundances greater than 1% are presented (Supplementary Fig. S6B). The most abundant genera included *Blautia* (11.1%), *Butyrivibrio* (10.9%), *Faecalibacterium* (10.4%), *Phocaeicola* (10.3%), *Bacteroides* (6%), and *Bifidobacterium* (5.3%). At the species level, the top 26 species with relative abundances greater than 1% ware presented (Supplementary Fig. S6C). The five most abundant species were *Butyrivibrio fibrisolvens* (10.9%), *Faecalibacterium prausnitzii* (10.4%), *Phocaeicola vulgatus* (7.5%), *Blautia schinkii* (5.2%), and *Blautia wexlerae* (4.3%).

**Fig. 6:**
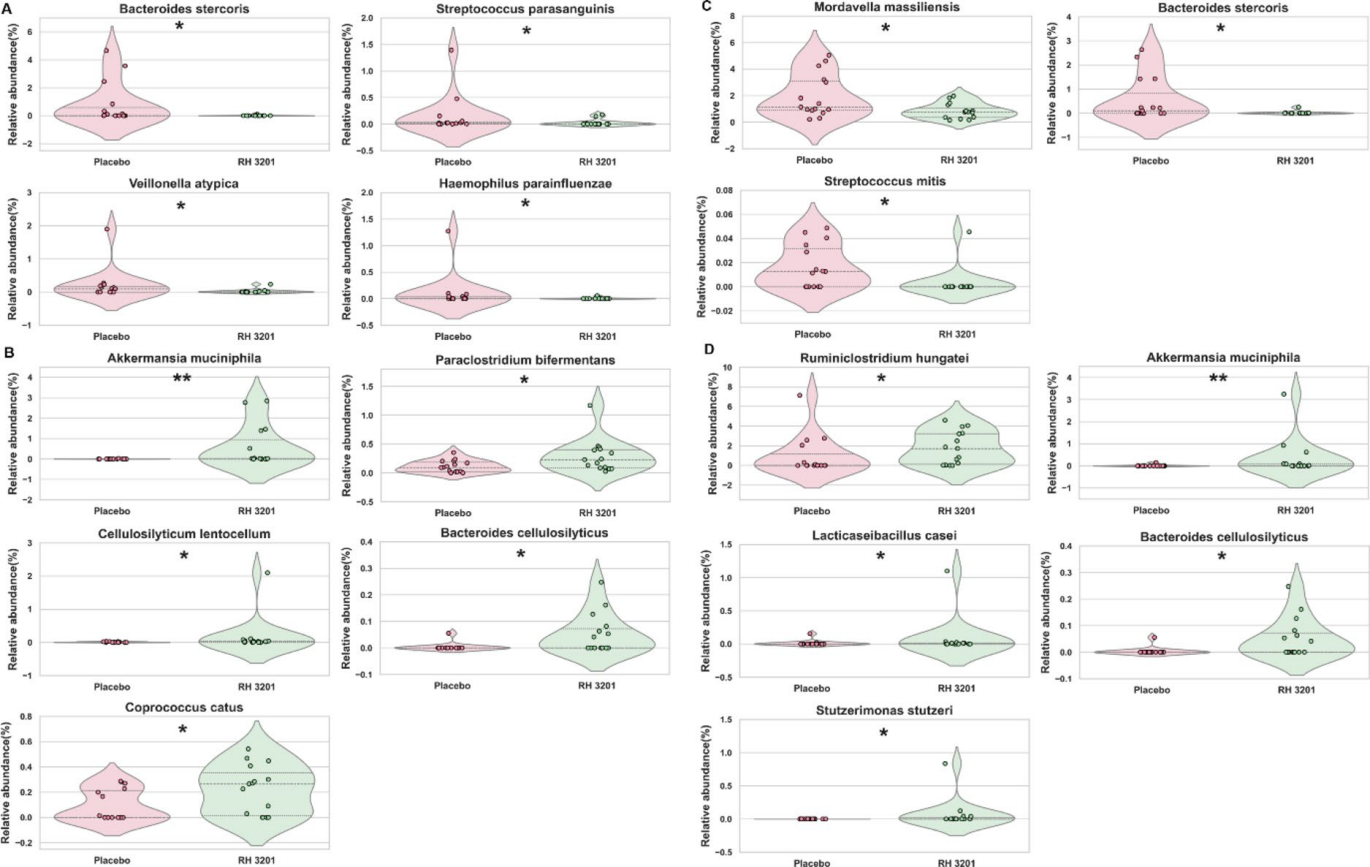
Enrichment analysis at the species level between the placebo and RH 3201 group using linear discriminant effect size (LEfSe) analysis. Species differentially abundant in (**A**) placebo and (**B**) RH 3201 groups at 4-week, and (**C**) placebo and (**D**) RH 3201 at 8-week. Significant differences between the groups are indicated as * (*p* < 0.05) and ** (*p* < 0.01) using Mann Whitney U-test.

Next, linear discriminant analysis effect size (LefSe) analysis was employed to identify specific bacteria that were differentially abundant in the microbial clusters of the placebo and RH 3201 groups (Fig. 6). At 4 weeks of intervention, the placebo group was enriched in *Bacteroides stercoris*, *Veillonella atypica*, *Streptococcus parasanguinis*, and *Haemophilus parainfluenzae*, whereas the RH 3201 group was enriched in *Akkermansia muciniphila*, *Paraclostridium bifermentans*, *Cellulosilyticum lentocellum*, and *Bacteroides cellulosilyticus* (Fig. 6A and B). The placebo group at 8 weeks of intervention showed differential abundance of *Mordavella massiliensis*, *B. stercoris*, and *Streptococcus mitis* (Fig. 6C). *Rumiclostridium hungatei*, *(A) muciniphila*, *Lacticaseibacillus casei*, *Stutzerimonas stutzeri*, and *(B) cellulosilyticus* were differentially abundant in the RH 3201 group at week 8 (Fig. 6D). Interestingly, *(A) muciniphila* and *(B) cellulosilyticus* were found to be enriched in both the intervention weeks of 4 and 8 in the RH 3201 group. In summary, the administration of RH 3201 led to a change in the fecal microbiome composition, specifically increasing abundance of *(A) muciniphila* and *(B) cellulosilyticus*.

### RH 3201 modulates fecal metabolome profiles

**Fig. 7:**
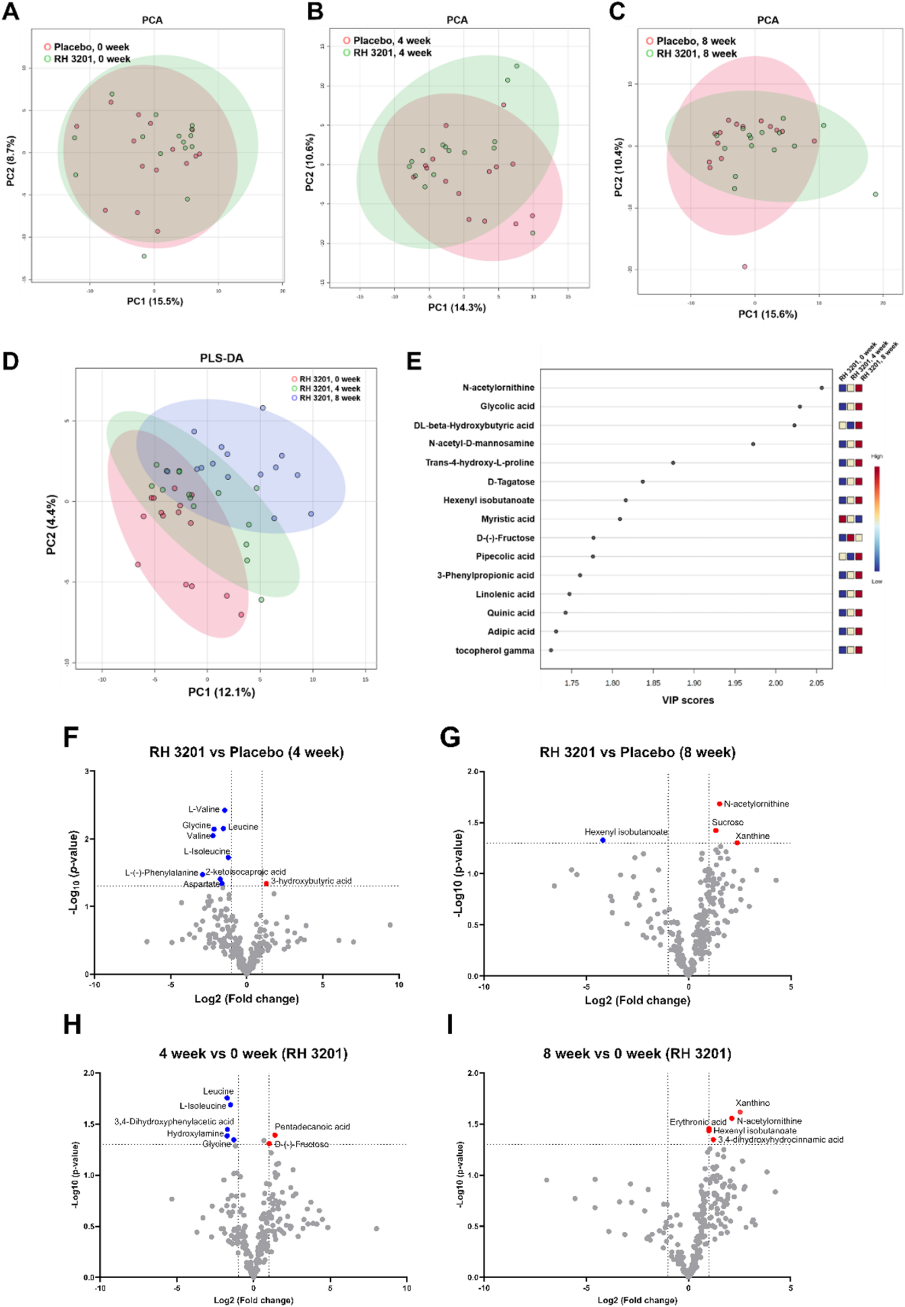
Multivariate (**A**–**E**) and univariate (**F**–**I**) analyses of fecal metabolome over time of intervention in the placebo and RH 3201 groups. (**A**–**C**) Unsupervised principal component analysis (PCA) with an auto-scaling method. (**D**) Supervised partial least squares discriminant analysis (PLS-DA) with an auto-scaling method. (**E**) Discriminant metabolic features identified according to the Variable Importance in Projection (VIP) scores. The 15 most important metabolites with value of VIP score > 1.5 are reported. (**F**–**I**) Volcano plot analyses of differentially abundant fecal metabolites over time of intervention in the placebo and RH 3201 groups. RH 3201 versus placebo at 4-week (**F**) and 8-week (**G**). Changes on metabolite abundances of RH 3201 at 4-week (**H**) and 8-week (**I**) compared to the 0-week control. Blue and red dots represent significant decreases and increases in metabolites, respectively (*p* < 0.05). Gray dots represent all other metabolites identified in the dataset of which relative concentrations did not change significantly between the two groups.

In order to refine the effects of RH 3201 on improved clinical outcomes, we further analyzed fecal metabolites in the placebo and RH 3201 groups at 0, 4, and 8 weeks. A total of 237 metabolites were identified, including sugars, amino acids, fatty acids, organic acids, and polyamines, covering central carbon and amino acid metabolism (Supplementary Fig. S7). Based on unsupervised principal component analysis (PCA), a slight alteration in the metabolite profiles between the placebo and RH 3201 groups was observed (Fig. 7A–C). To discover possible biomarker candidates, metabolite profiles from the probiotics at different intervention periods were analyzed using supervised partial least squares-discriminant analysis (PLS-DA) (Fig. 7D). Compared with the beginning of the intervention (0 week), fecal metabolites in the RH 3201-administered patients shifted along with the intervention stages. N-acetylornithine, glycolic acid, and beta-hydroxybutyric acid showed higher VIP (variable importance for projection) scores, indicating measures of individual metabolite importance in the PLS-DA model (Fig. 7E)^35–38^. 

**Fig. 8:**
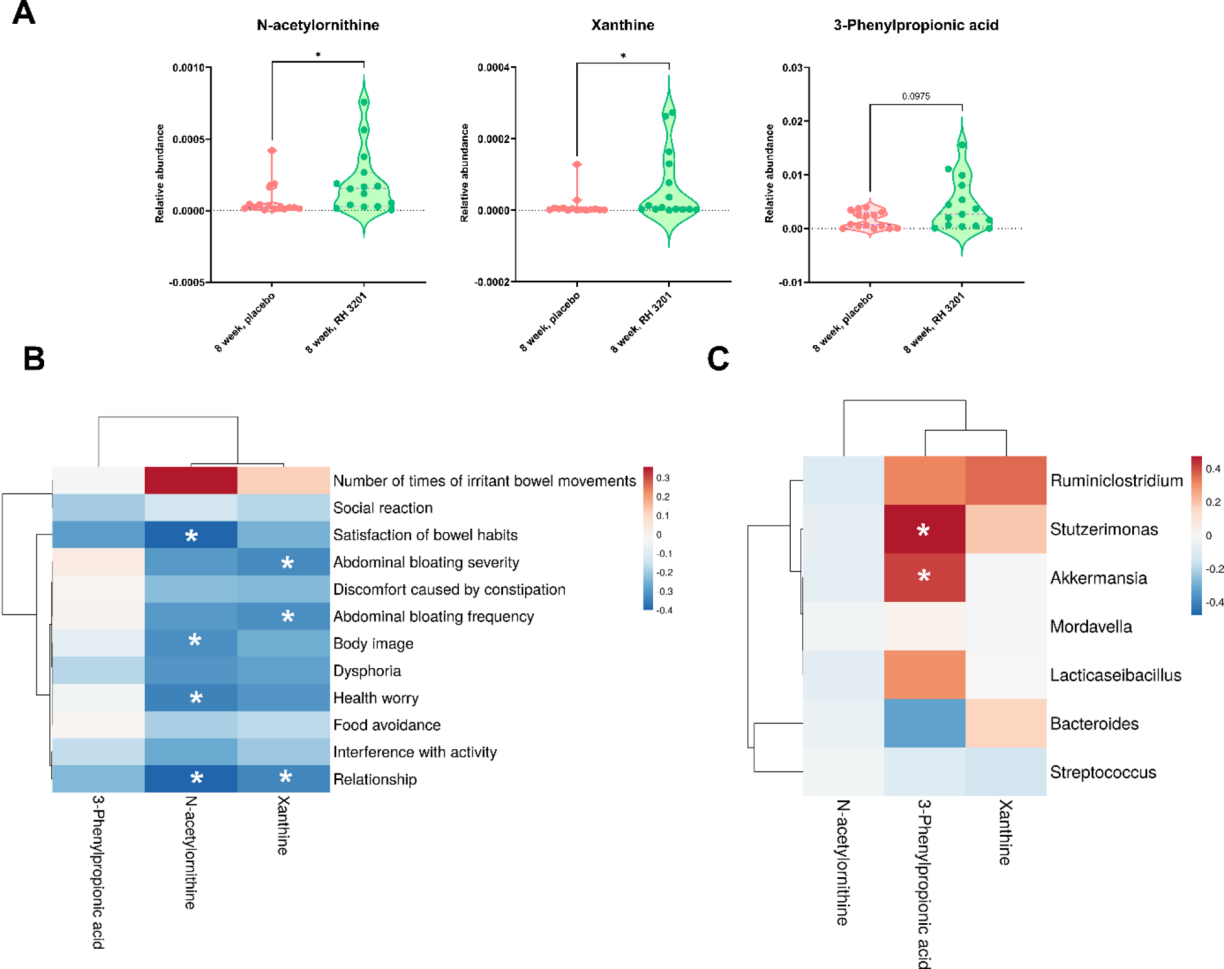
Relative abundances of specific metabolites highly abundant in the RH 3201 groups (**A**) and Spearman correlation analyses of clinical parameters (**B**) and fecal microbiome (**C**) with the metabolites. Metabolites differentially abundant in RH 3201 during the 8-weeks of intervention compared to the placebo group were selected (N-acetylornithine, xanthine, and 3-phenylpropionic acid), and their relative abundance was compared at 8-week (**A**). Significant differences between the groups are indicated as * (*p* < 0.05) using Mann Whitney U-test. For Spearman correlation, each row represents selected clinical questionnaires (**B**) and bacterial genera differentially abundant in RH 3201 compared to the placebo control at 8-week (**C**), and each column represents the selected metabolites. Red and blue are positive and negative correlation, respectively. Significant differences between the groups are indicated as * (*p* < 0.05).

Next, we performed pairwise t-tests of individual metabolites between the RH 3201 and placebo groups, as well as at different time points with probiotics administration (Fig. 7F and I). At 4 weeks, it was notable that several non-polar amino acids, including leucine and isoleucine, were significantly reduced in the RH 3201 group. However, differences in these metabolites disappeared at 8 weeks. Instead, by combining the results of univariate statistics and feature analysis represented as VIP scores, we identified specific metabolites, including N-acetylornithine, xanthine, and 3-phenylpropionic acid, that were significantly altered during the 8 weeks of RH 3201 administration (Supplementary Fig. S8 and Fig. 8A). In summary, these findings suggest that administration of RH 3201 altered the fecal metabolic profiles and promoted an increase in the abundance of specific metabolites in IBS-C patients.

### Specific factors of fecal microbiome and metabolome correlate with the clinical outcomes

The gut microbiome and its related microbial metabolites have been strongly linked to the pathophysiology of IBS^39–42^. To investigate potential interactions among clinical parameters, fecal microbiome, and metabolome, we performed Spearman correlation analyses at 8 weeks. Initially, we selected 12 clinical questionnaires that showed significant improvement with the administration of RH 3201, and calculated correlation coefficients with respect to the relative abundance of N-acetylornithine, xanthine, and 3-phenylpropionic acid (Fig. 8B). Notably, these three identified metabolites mostly showed negative correlation with the clinical outcomes, indicating an increase in their abundance with respect to the improvement of clinical symptoms in the RH 3201 group at 8 weeks compared with placebo. N-acetylornithine was found to significantly correlate with the improvement of clinical indications, including satisfaction of bowel habits, body image, healthy worry, and relationship, whereas xanthine was associated with abdominal bloating severity and abdominal bloating frequency.

Next, we performed the correlation analysis of the specific metabolites (N-acetylornithine, xanthine, and 3-phenylpropionic acid) with the metagenome profile to identify possible relationships between metabolite levels and specific bacterial abundances (Supplementary Fig. S9). Among the selected metabolites, 3-phenylpropionic acid had a particularly positive correlation with seven bacterial genera that significantly increased in RH 3201 at 8 weeks (Fig. 8C). Taking results, it is concluded that RH 3201 altered certain genera of gut microbiota and their metabolite production, which may affect the improvement of clinical symptoms.

## Discussion

RH 3201 is known for its functions in autoimmune regulation, anti-inflammation, and protein digestion^4,21,22^. In this study, we conducted a randomized, double-blind, placebo-controlled trial involving the administration of RH 3201 at a dose of 1 × 10^10^ CFU per day or placebo to patients with moderate IBS with constipation (IBS-C) to evaluate its therapeutic effect on IBS-C. Over the 8-week intervention period, bowel activities, IBS-SSS scores, including subscores related to abdominal bloating and dissatisfaction with bowel habits, and IBS-QOL, including dysphoria and health worry, were significantly improved in the RH 3201 group compared to the placebo group. Microbiome analysis indicated slight changes in bacterial diversity but a significant increase in specific strains such as *(A) muciniphila* and *(B) cellulosilyticus*. Metabolome analysis identified N-acetylornithine, xanthine, and 3-phenylpropionic acid as key fecal metabolites present in the RH 3201 group. These specific bacterial strains and metabolites showed a high correlation with clinical outcomes, suggesting that the interaction of RH 3201 with carbohydrate-degrading bacteria and the promotion of functional metabolite biosynthesis can be beneficial for intestinal health.

There is growing interest in the beneficial effects of probiotics on the treatment of IBS-C. Possible mechanisms of probiotic action in IBS-C have been suggested to include inhibition of pathogen binding, enhanced barrier function, anti-inflammation, alterations in visceral hypersensitivity, and modification of gut microbiota^20^. In addition to alleviating IBS-C symptoms, quality of life was significantly improved in the probiotic group compared to the placebo, particularly in emotional well-being and social functioning. Generally, IBS patients have a higher risk of psychiatric comorbidities than healthy individuals. Some probiotics are known to affect psychiatric functions by communicating via the bacteria-gut-brain axis^43–45^. In a placebo-controlled trial, *Bifidobacterium longum* NCC3001 was shown to reduce depression and increase QOL in IBS patients, associated with changes in brain activation patterns^46^. A meta-analysis of the effect of probiotics on QOL indicated that probiotic therapy improved QOL, although there were no significant differences in anxiety and depression between the placebo and probiotic groups^47^.

In the current study, RH 3201 improved IBS-C-related symptoms and IBS-QOL in patients with IBS-C. RH 3201 has been proven to regulate hypersensitive immune reactions, provide anti-inflammatory benefits, exhibit anti-pathogenic activity, and enhance protein digestibility^4,21,22^. Oral administration of RH 3201 in mice showed reduced mast cell numbers and production of immune-modulating cytokines, including interleukin-4 (IL-4) and regulatory T cytokine interleukin-10 (IL-10)^21^. In addition, treatment of RAW264.7 macrophages with cell-free supernatant of RH 3201 reduced the expression of pro-inflammatory cytokines, including tumor necrosis factor alpha (TNF-α) and interleukin-6 (IL-6), as well as genes involved in the production of reactive oxygen species, including *COX-2* and *iNOS*^22^. Furthermore, the supernatant inhibited the growth of intestinal pathogens, including *Bacillus cereus*, *Enterococcus faecalis*, and *Salmonella* Typhimurium^22^. Based on these findings, it is speculated that RH 3201 may function in immune modulation and bacterial competition, particularly against intestinal pathogens, resulting in the amelioration of the pathophysiology of IBS-C.

A recent study observed a higher relative abundance of methane- and hydrogen-producing bacteria in IBS-C patients^48,49^. Methane and hydrogen are known to decrease the amplitude of contractions and slow peristalsis, leading to constipation. Considering the competitive nature of RH 3201 with gut pathogens, it is speculated that this probiotic strain may inhibit methanogens and hydrogen producers, resulting in reduced discomfort and dissatisfaction with bowel habits.

The pathophysiology of IBS is still largely undefined, but cumulative evidence suggests a contribution from the gut microbiota^50^. Studies on the fecal microbiota of IBS patients have shown that it differs significantly from that of healthy subjects^51^, and changes in the diversity of the gut microbiome are associated with IBS^52^. Our results show that the administration of RH 3201 reshapes the fecal bacterial community, particularly modifying the relative composition of specific strains, including *(A) muciniphila* and *(B) cellulosilyticus*.

*A. muciniphila* is a commensal bacterium, recognized for its mucin-layer-degrading activity^53^. Metagenomic studies have identified its inverse correlation with human diseases such as inflammatory bowel disease^54^, obesity^55^, and diabetes^56^, potentially due to its immunomodulatory activities. Accumulating evidence suggests that the administration of *lactobacilli* increases the abundance of *A. muciniphila*^57–59^. For example, *L. paragasseri* has been shown to improve depression-like behavior and increase *A. muciniphila* in mice^58^. Similarly, *L. acidophilus* LA5 attenuated obesity in mice and increased *A. muciniphila*^59^. Although the specific mechanisms underlying the interrelationship between probiotic strains and *A. muciniphila* still need to be elucidated, their symbiotic effects on human disease prevention are becoming increasingly evident.

The consumption of large amounts of insoluble dietary fiber often exacerbates symptoms in IBS patients^60^. It is known that a low FODMAP (fermentable oligosaccharides, disaccharides, monosaccharides, and polyols) diet may ameliorate IBS symptoms, particularly bloating and colonic distention^61^. This diet’s restriction of multiple fermentable components primarily prevents the augmentation of small intestinal water and colonic gas production caused by gut microbiota, suggesting that the breakdown of insoluble oligosaccharides could be beneficial. *B. cellulosilyticus*, a strictly anaerobic cellulolytic bacterium firstly isolated from human feces, is capable of degrading various types of polysaccharides^62^. Several studies have demonstrated an inverse relationship between *B. cellulosilyticus* and inflammatory bowel disease, mitigating symptoms via the regulation of Treg cells and fiber degradation^63,64^. The administration of RH 3201 is hypothesized to promote the growth of *B. cellulosilyticus* through an unidentified mechanism, potentially resulting in the degradation of fibers and a reduction in IBS-C symptoms such as bloating.

*Lactobacilli* form significant populations in the human intestinal tract, especially in the small intestine^65^. Bacteria commonly found in the small intestine include *Lactobacillus*, *Clostridium*, *Streptococcus*, and *Bacteroides*^66^. These bacteria play roles in nutrient digestion, by-product formation such as hydrogen sulfide and hydrogen gas, and the synthesis of micronutrients in the small intestine^66^. Although the current research only measured the human fecal microbiome, changes in the microbial composition also reflect a part of the small intestinal community and suggest that RH 3201 may promote the growth of these small intestinal bacteria, particularly those participating in nutrient digestion such as *B. cellulosilyticus*.

In the small intestine, enteroendocrine cells are key sensors of luminal nutrients and gut microbiota, playing roles in mucosal immunity, gut barrier function, visceral hyperalgesia, and intestinal motility^67^. Patients with IBS generally have abnormal densities of enteroendocrine cells^68^. It has been suggested that a low FODMAP diet and gut microbiota modify the densities of enteroendocrine cells in the gastrointestinal tract of IBS patients towards the densities measured in healthy controls^69,70^. Although a direct relationship between *Lactobacillus* and enteroendocrine cells has not been reported yet, evidence from the alteration of the gut microbial community in this study suggests a possible association of RH 3201 with the gut mucosal environment and luminal changes in nutrients affecting the functions of enteroendocrine cells.

In the current study, we observed an increase in the levels of N-acetylornithine, xanthine, and 3-phenylpropionic acid in the fecal sample of patients administered RH 3201. N-acetylornithine is an intermediate in arginine and proline metabolism, that can be found in many living systems, ranging from bacteria to humans. A reduced abundance of this metabolite has been observed in humans with psychiatric disorders^71^. Yang et al. reported that the level of N-acetylornithine decreased in patients with major depressive disorders compared to healthy controls^36^. Similarly, the characterization of the serum metabolome in patients with functional constipation showed a significant reduction in N-acetylornithine^72^. Although the specific mechanism of this metabolite needs to be elucidated, it may stimulate gastrointestinal motility^73^ or improve psychiatric function.

Purine metabolites, including xanthine, act as chemical messengers in purinergic signaling, coordinating many physiological processes involved in neuropathy and inflammation^74^. *Lactobacilli* have been found to inhibit xanthine oxidase, an enzyme that catalyzes the production of uric acid from xanthine, thereby helping to prevent hyperuricemia^75^. In several studies, the activity of xanthine oxidase was found to be significantly higher in IBS patients than in controls, likely due to lipid peroxidation^76,77^. Administration of RH 3201 may lower the enzyme activity associated with immunological amelioration in patients, and fecal xanthine level could be an effective biomarker. 3-Phenylpropionic acid is a gut microbial metabolic product of L-phenylalanine. Gut microbiota-derived 3-phenylpropionic acid is known to promote intestinal epithelial barrier function^78^ and alleviate hepatotoxicity^79^. Although the specific bacterial strains producing this metabolite are still unclear, our results revealed that several strains such as *A. muciniphila* would be associated with 3-phenylpropionic acid. As an increasing number of studies are focusing on the importance of 3-phenylpropionic acid in disease prevention and treatment, further studies on RH 3201 and its metabolite production are required.

In conclusion, this study indicates that the probiotic RH 3201 may contribute to the improvement of IBS-C symptoms by modulating fecal microbiota and their metabolite production. However, this study has limitations in that the clinical outcomes were primarily based on the questionnaires; hence, physical assessments such as colonoscopy or stool tests would support the findings. In addition, further studies are necessary to elucidate a causal relationship and clarify the mechanism of action of this probiotic strain. With additional supporting mechanistic studies, RH 3201 has the potential to be a promising therapeutic candidate for treating IBS, offering valid functions and effective applications.

## Electronic supplementary material

Below is the link to the electronic supplementary material.

Supplementary Material 1

## Data Availability

The raw reads were deposited in the NCBI Sequence Read Archive (SRA) database (Accession Numbers: 27193317-27193406).
