# Supplementary material for: Effect of Lacticaseibacillus rhamnosus IDCC 3201 on irritable bowel syndrome with constipation: a randomized, double-blind, and placebo-controlled trial
Source: Sci Rep. 2024 Sep 27;14:22384. doi: 10.1038/s41598-024-72887-x (PMC11437119; doi:10.1038/s41598-024-72887-x)

**Effect of *Lacticaseibacillus rhamnosus* IDCC 3201 on irritable bowel syndrome with constipation: A randomized, double-blind, and placebo-controlled trial**

Hyeji Kwon^1#^, Eoun Ho Nam^2,3#^, Hayoung Kim^4#^, Haneul Jo^2,3^, Won Yeong Bang^4^, Minjee Lee^4^, Hyeonmin Shin^1^, Dana Kim^1^, Jeongho Kim^5^, Hyejin Kim^5^, Jongkyun Lee^6^, Young Hoon Jung^7,8^, Jungwoo Yang^4,9^*, Daeyoun David Won^6^*, Minhye Shin^2,3^*

^1^ Cancer Genomic Research Institute, Immunology Laboratory, Seoul Song Do Colorectal Hospital, Seoul, 04597, Republic of Korea.

^2^ Department of Microbiology, College of Medicine, Inha University, Incheon, 22212, Republic of Korea

^3^ Department of Biomedical Science, Program in Biomedical Science and Engineering, Inha University, Incheon, 22212, Republic of Korea

^4^ Ildong Bioscience, Pyeongtaek-si, Gyeonggi-do, 17957, Republic of Korea

^5^ Digestive Endoscopic Center, Seoul Song Do Colorectal Hospital, Seoul, 04597, Republic of Korea

^6^ Department of Surgery, Pelvic Floor Center, Seoul Song Do Colorectal Hospital, Seoul, 04597, Republic of Korea

^7^ School of Food Science and Biotechnology, Kyungpook National University, Daegu, 41566, Republic of Korea

^8^ Institute of Fermentation Biotechnology, Kyungpook National University, Daegu, 41566, Republic of Korea

^9^ Department of Microbiology, Dongguk University College of Medicine, 123 Dongdae-ro, Gyeongju, 38066, Republic of Korea

^#^ Hyeji Kwon, Eoun Ho Nam, and Hayoung Kim contributed equally to this work.

* Corresponding authors: Jungwoo Yang ([mqbssjy2@gmail.com](mailto:mqbssjy2@gmail.com)), Daeyoun David Won ([greatsongdo@gmail.com](mailto:greatsongdo@gmail.com)), Minhye Shin ([mhshin@inha.ac.kr](mailto:mhshin@inha.ac.kr))

# **Supplementary information**

# **Supplementary Table S1.** Composition of investigational products

|  |  | (Composition: %) |
| --- | --- | --- |
| **Raw material** | **Placebo** | **RH 3201** |
| *Lactobacillus rhamnosus* IDCC 3201 | - | 33.334 |
| Maltodextrin | 97.935 | 64.666 |
| Silicon dioxide | 1.000 | 1.000 |
| Orange color powder | 0.065 | - |
| Magnesium stearate | 1.000 | 1.000 |
| Total | 100 | 100 |

# **Supplementary Table S2.** Schedule for data collection

| **Item** | **Visit** | | | |
| --- | --- | --- | --- | --- |
|  | **1^(-2 week)^** | **2^(0 week)^** | **3^(4 week)^** | **4^(8 week)^** |
| Informed consent | ● |  |  |  |
| Demographic information | ● |  |  |  |
| Medical and operational history | ● |  |  |  |
| Medication and dietary supplement use | ● | ● | ● | ● |
| Anthropometric measurement | ● |  |  | ● |
| Vital signs | ● |  |  | ● |
| Eligibility assessment | ● |  |  |  |
| Random allocation |  | ● |  |  |
| Supply with test/placebo food |  | ● | ● |  |
| Questionnaire |  | ● | ● | ● |
| Stool sampling |  | ● | ● | ● |
| Compliance |  |  | ● | ● |
| Adverse events |  |  | ● | ● |

**Supplementary Table S3.** Baseline characteristics of participants

| **Characteristics** | **Placebo**  **(n=15)** | **RH 3201**  **(n=15)** | ***p*-value** |
| --- | --- | --- | --- |
| Age (y) | 37.1 ± 13.7 | 36.4 ± 13.6 | 0.90 |
| Gender (M/F) | 6 / 9 | 3 / 12 | 0.25 |
| Height (cm) | 163.9 ± 8.5 | 164.6 ± 6.5 | 0.80 |
| Weight (kg) | 65.7 ± 15.9 | 58.9 ± 11.4 | 0.19 |
| Current smoking | 0.1 ± 0.4 | 0 ± 0 | 0.15 |
| Drinking alcohol | 0.4 ± 0.5 | 0.5 ± 0.5 | 0.72 |
| Light-intensity physical activity | 3.3 ± 1.7 | 3.3 ± 2.4 | 0.93 |
| Moderate-Intensity physical activity | 2.9 ± 2.1 | 3.1 ± 2.1 | 0.73 |
| Sleep hour | 6.1 ± 1.0 | 6.7 ± 1.3 | 0.19 |
| Systolic blood pressure (mmHg) | 122.9 ± 15.3 | 116.5 ± 13.1 | 0.22 |
| Diastolic blood pressure (mmHg) | 70.5 ± 13.9 | 65.0 ± 11.18 | 0.24 |
| Pulse (min) | 76.1 ± 11.5 | 79.9 ± 13.3 | 0.40 |

**Supplementary Table S4.** Characteristics of participant before and after prescription

|  | **Placebo** | | | **RH 3201** | | |
| --- | --- | --- | --- | --- | --- | --- |
| **Characteristics** | **0 week** | **8 week** | ***p*-value** | **0 week** | **8 week** | ***p*-value** |
| Height (cm) | 164.6 ± 6.5 | 164.5 ± 6.8 | 0.950 | 163.9 ± 8.5 | 164.5 ± 6.8 | 0.970 |
| Weight (kg) | 58.9 ± 11.4 | 59.5 ± 12.2 | 0.893 | 65.7 ± 15.9 | 66.1 ± 16.2 | 0.944 |
| Systolic blood pressure (mmHg) | 116.5 ± 13.1 | 116.7 ± 13.7 | 0.956 | 122.9 ± 15.3 | 123.2 ± 16.2 | 0.963 |
| Diastolic blood pressure (mmHg) | 65.0 ± 11.2 | 70.9 ± 13.9 | 0.208 | 70.5 ± 13.9 | 74.0 ± 11.3 | 0.459 |
| Pulse (min) | 79.9 ± 13.3 | 77.1 ± 9.5 | 0.514 | 76.1 ± 11.5 | 76.8 ± 9.5 | 0.850 |

**Supplementary Table S5.** Assessment of bowel activities in participants

| **Item** | **0 week** | | | | **4 week** | | | | **8 week** | | |
| --- | --- | --- | --- | --- | --- | --- | --- | --- | --- | --- | --- |
|  | **Placebo** | **RH 3201** | ***p*-value** | **Placebo** | | **RH 3201** | ***p*-value** | **Placebo** | | **RH 3201** | ***p*-value** |
| Number of bowel movement  per week | 3.5 ± 3.4 | 3.1 ± 1.8 | 0.736 | 4.2 ± 3.0 | | 3.9 ± 2.4 | 0.788 | 4.5 ± 4.0 | | 4.2 ± 1.5 | 0.811 |
| Defecation time | 2.5 ± 0.9 | 2.3 ± 0.7 | 0.379 | 2.5 ± 1.1 | | 2.1 ± 1.0 | 0.381 | 2.4 ± 1.0 | | 1.9 ± 0.7 | 0.147 |
| Amount of feces | 2.7 ± 0.9 | 2.4 ± 1.1 | 0.463 | 2.6 ± 0.8 | | 2.5 ± 0.9 | 0.836 | 2.6 ± 0.9 | | 2.5 ± 0.9 | 0.843 |
| Number of times of irritant bowel  movements | 6.3 ± 2.1 | 5.2 ± 2.1 | 0.157 | **5.9 ± 2.5** | | **3.9 ± 2.1** | **0.026** | **5.0 ± 2.5** | | **3.0 ± 1.6** | **0.013** |
| Number of times when bowel  movements felt incomplete | 6.7 ± 1.5 | 6.3 ± 2.7 | 0.561 | 6.2 ± 2.1 | | 5.2 ± 2.7 | 0.248 | 4.9 ± 2.5 | | 3.3 ± 2.6 | 0.10 |
| Shape of the feces | 2.9 ± 1.4 | 2.8 ± 1.2 | 0.890 | 3.6 ± 1.5 | | 3.5 ± 0.9 | 0.776 | 2.9 ± 1.4 | | 3.4 ± 1.2 | 0.261 |
| Number of times of abdominal  pains before bowel movements | 5.5 ± 3.1 | 5.3 ± 3.3 | 0.866 | 4.8 ± 2.6 | | 4.8 ± 3.0 | 0.974 | 4.2 ± 2.5 | | 3.4 ± 2.5 | 0.390 |
| Number of times of abdominal  pains during bowel movements | 4.4 ± 2.7 | 5.2 ± 2.9 | 0.441 | 4.0 ± 2.7 | | 3.6 ± 2.7 | 0.711 | 3.8 ± 2.7 | | 3.3 ± 2.5 | 0.607 |
| Degree of abdominal pain | 4.3 ± 2.4 | 4.7 ± 2.6 | 0.721 | 4.2 ± 2.1 | | 3.5 ± 1.9 | 0.342 | 3.6 ± 2.3 | | 2.8 ± 1.7 | 0.271 |
| Amount of gas | 6.7 ± 2.4 | 6.8 ± 2.7 | 0.887 | 5.6 ± 2.6 | | 5.5 ± 2.5 | 0.916 | 5.4 ± 2.6 | | 4.1 ± 2.8 | 0.203 |
| Discomfort after bowel movements | 5.5 ± 2.5 | 5.3 ± 2.4 | 0.770 | **5.3 ± 2.3** | | **3.3 ± 1.8** | **0.016** | 4.2 ± 2.7 | | 2.9 ± 2.4 | 0.174 |
| Discomfort caused by constipation | 5.8 ± 2.1 | 6.0 ± 2.2 | 0.802 | **4.9 ± 2.2** | | **3.1 ± 1.9** | **0.022** | **4.3 ± 2.5** | | **2.3 ± 2.2** | **0.03** |

**Supplementary Table S6.** Assessment of IBS-SSS (Severity Scoring System) scores in participants

| **IBS-SSS Score** | **0 week** | | | **4 week** | | | **8 week** | | |
| --- | --- | --- | --- | --- | --- | --- | --- | --- | --- |
|  | **Placebo** | **RH 3201** | ***p*-value** | **Placebo** | **RH 3201** | ***p*-value** | **Placebo** | **RH 3201** | ***p*-value** |
| Severity of abdominal pain | 47.3 ± 26.0 | 40.3 ± 26.1 | 0.468 | 46.0 ± 17.7 | 36.3 ± 23.3 | 0.211 | 40.0 ± 23.2 | 25.0 ± 20.9 | 0.073 |
| Frequency abdominal pain | 30.3 ± 19.3 | 36.0 ± 26.4 | 0.508 | 34.7 ± 18.9 | 27.3 ± 19.4 | 0.303 | 25.7 ± 12.9 | 22.7 ± 20.5 | 0.636 |
| Severity of abdominal bloating | 59.7 ± 20.7 | 57.3 ± 20.6 | 0.760 | 46.3 ± 20.4 | 46.3 ± 28.9 | 1.000 | **48.3 ± 21.4** | **25.0 ± 28.3** | **0.017** |
| Dissatisfaction of bowel habits | 74.3 ± 14.5 | 62.0 ± 21.3 | 0.074 | **65.7 ± 7.5** | **51.3 ± 21.8** | **0.023** | **68.0 ± 15.3** | **44.7 ± 26.1** | **0.006** |
| Interference with quality of life | 65.0 ± 11.8 | 57.3 ± 20.4 | 0.219 | **58.0 ± 16.7** | **37.0 ± 20.9** | **0.005** | **55.3 ± 17.8** | **29.0 ± 18.8** | **< .001** |
| Total | 276.7 ± 60.4 | 253 ± 84.4 | 0.385 | 250.7 ± 56.8 | 198.3 ± 97.6 | 0.084 | **237.3 ± 73.5** | **146.3 ± 94.4** | **0.006** |

**Supplementary Table S7.** Assessment of IBS-QOL (Quality Of Life) in participants

| **IBS-QOL** | **0 week** | | | | **4 week** | | | **8 week** | | |
| --- | --- | --- | --- | --- | --- | --- | --- | --- | --- | --- |
|  | **Placebo** | **RH 3201** | ***p*-value** | **Placebo** | | **RH 3201** | ***p*-value** | **Placebo** | **RH 3201** | ***p*-value** |
| Dysphoria | 20.3 ± 5.9 | 17.5 ± 6.2 | 0.216 | **18.1 ± 6.0** | | **13.5 ± 4.0** | **0.021** | **18.2 ± 8.0** | **10.6 ± 3.0** | **0.002** |
| Interference with activity | 17.2 ± 5.5 | 14.5 ± 6.1 | 0.207 | 16.1 ± 6.1 | | 12.8 ± 4.5 | 0.106 | **16.8 ± 7.4** | **10.5 ± 3.7** | **0.006** |
| Body image | 10.0 ± 3.8 | 8.9 ± 3.0 | 0.397 | 9.4 ± 3.9 | | 8.1 ± 3.0 | 0.298 | **9.4 ± 4.3** | **6.1 ± 2.2** | **0.014** |
| Health worry | 9.1 ± 2.1 | 8.3 ± 2.6 | 0.366 | **9.2 ± 2.6** | | **6.7 ± 2.4** | **0.012** | **8.2 ± 3.0** | **4.9 ± 2.1** | **0.002** |
| Food avoidance | 8.7 ± 3.4 | 7.4 ± 3.0 | 0.288 | 8.6 ± 3.5 | | 6.7 ± 2.0 | 0.084 | **7.8 ± 3.3** | **5.2 ± 1.9** | **0.014** |
| Social reaction | 8.7 ± 3.0 | 8.1 ± 3.3 | 0.646 | 8.1 ± 3.4 | | 7.0 ± 2.4 | 0.326 | **8.8 ± 4.0** | **5.1 ± 1.9** | **0.003** |
| Sexual | 3.4 ± 1.8 | 3.1 ± 1.5 | 0.660 | 3.3 ± 1.6 | | 2.8 ± 1.2 | 0.318 | 3.5 ± 2.3 | 2.4 ± 0.7 | 0.102 |
| Relationship | 6.3 ± 2.1 | 5.8 ± 2.1 | 0.493 | 6.1 ± 2.7 | | 4.9 ± 1.6 | 0.145 | **6.8 ± 2.8** | **3.9 ± 1.6** | **0.002** |
| Total | 83.7 ± 22.6 | 73.7 ± 24.1 | 0.251 | 78.9 ± 26.4 | | 62.6 ± 16.9 | 0.055 | **79.5 ± 33.0** | **48.7 ± 14.5** | **0.003** |

**Supplementary Fig S1.** PCR of fecal samples from the RH 3201 (A) and placebo (B) groups at 0, 4, 8 weeks of administration and relative abundance of the genus (C).


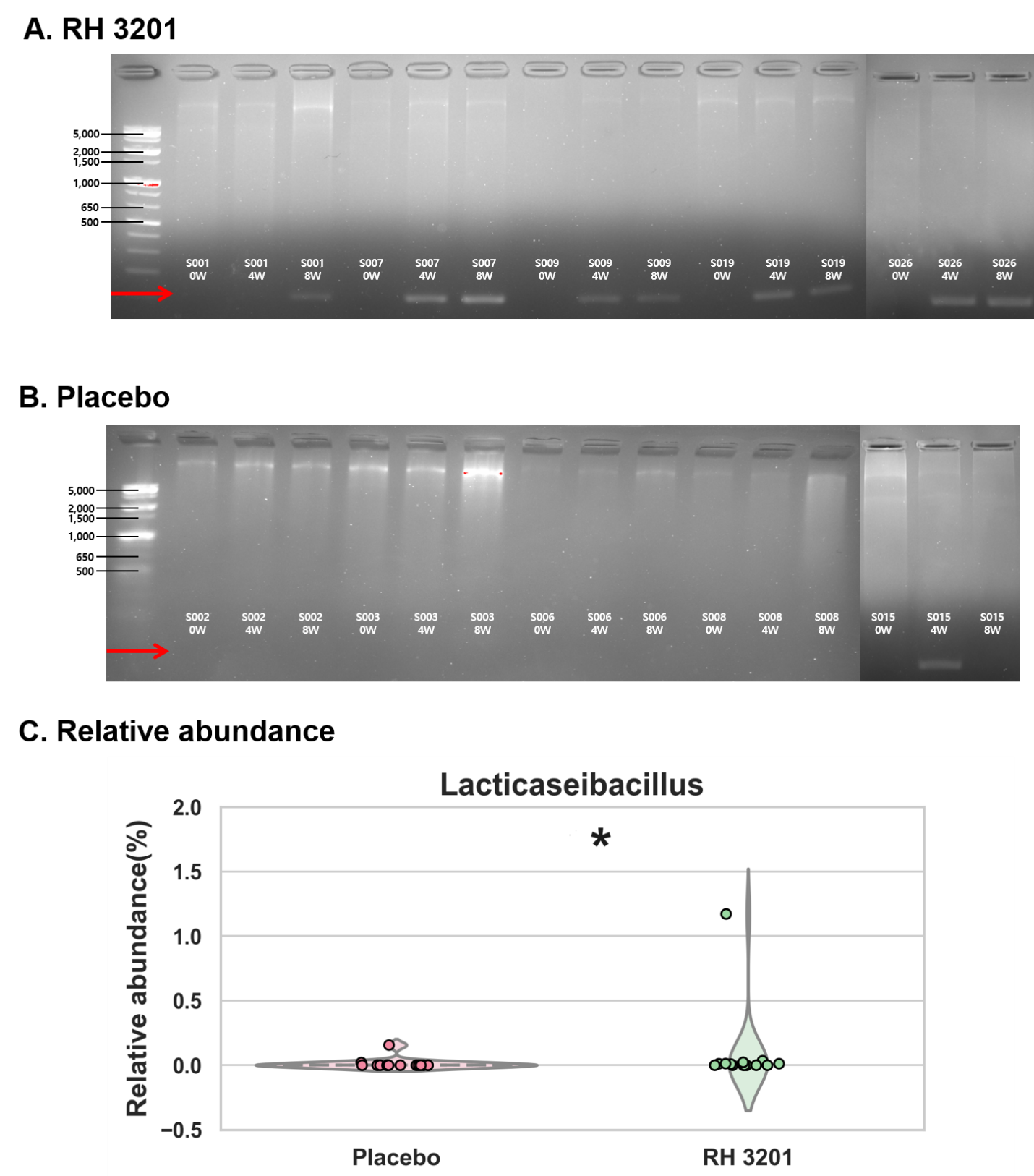


# **Supplementary Fig S2.** Assessment of bowel activities in participants by RH 3201. (A) Number of times of irritant bowel movements. (B) Discomfort after bowel movements. (C) Discomfort caused by constipation. Data were expressed as mean ± standard deviation. Significant differences compared to the 0-week group are indicated as * (p < 0.05), ** (p < 0.01) and *** (p < 0.001) using repeated measures ANOVA with Tukey as a post-hoc analysis.


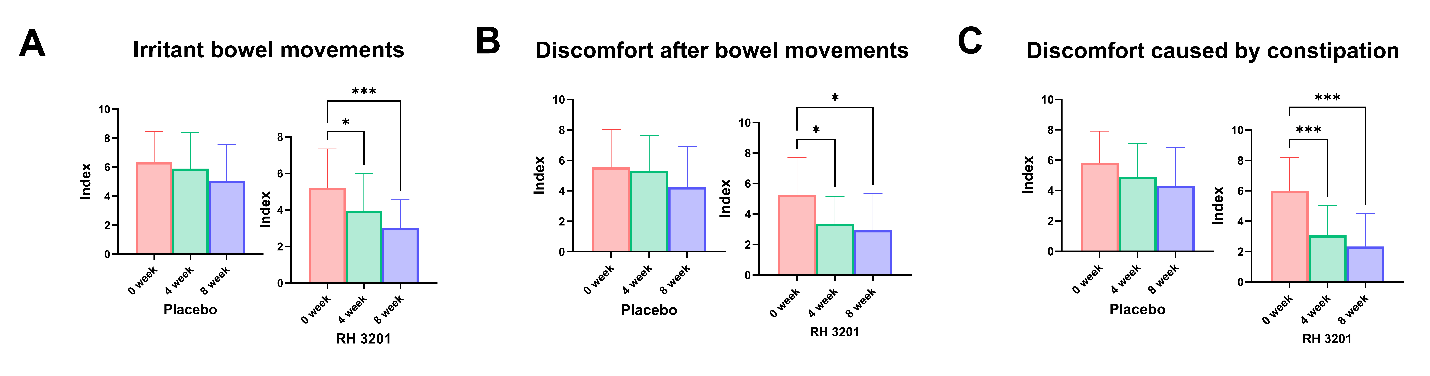


# **Supplementary Fig S3.** Assessment of IBS-SSS scores in participants by RH 3201. (A) Total IBS-SSS score. (B) Severity of abdominal bloating. (C) Frequency of abdominal bloating. (D) Dissatisfaction of bowel habits. Data were expressed as mean ± standard deviation. Significant differences compared to the 0-week group are indicated as * (p < 0.05), ** (p < 0.01) and *** (p < 0.001) using repeated measures ANOVA with Tukey as a post-hoc analysis.


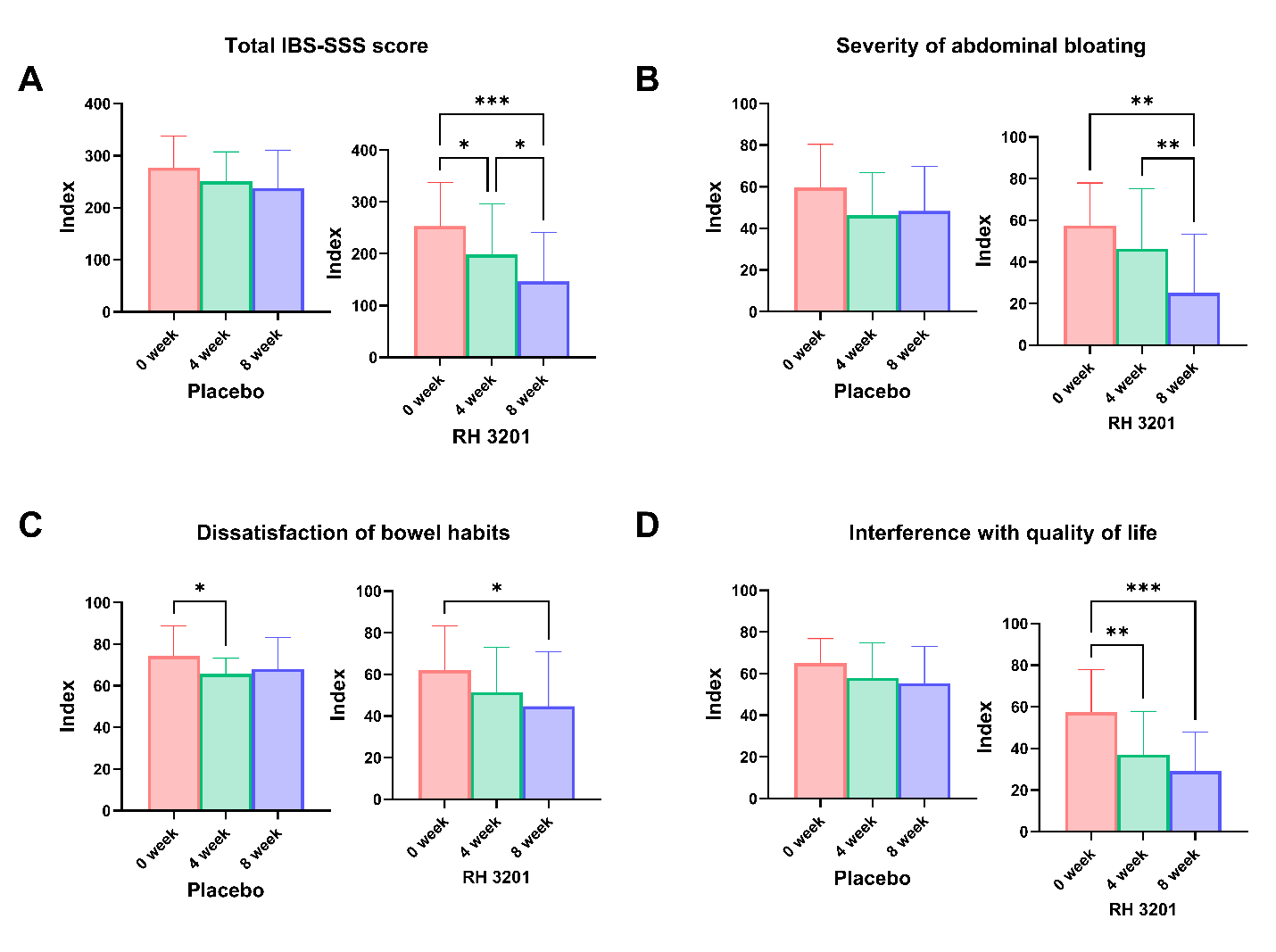


# **Supplementary Fig S4.** Assessment of IBS-QOL (Quality Of Life) in participants by RH 3201. (A) Dysphoria. (B) Interference with activity. (C) Body image. (D) Health worry. (E) Food avoidance. (F) Social reaction. (G) Relationship. (H) Total QOL score. Data were expressed as mean ± standard deviation. Significant differences compared to the 0-week group are indicated as * (p < 0.05) and ** (p < 0.01) using repeated measures ANOVA with Tukey as a post-hoc analysis.


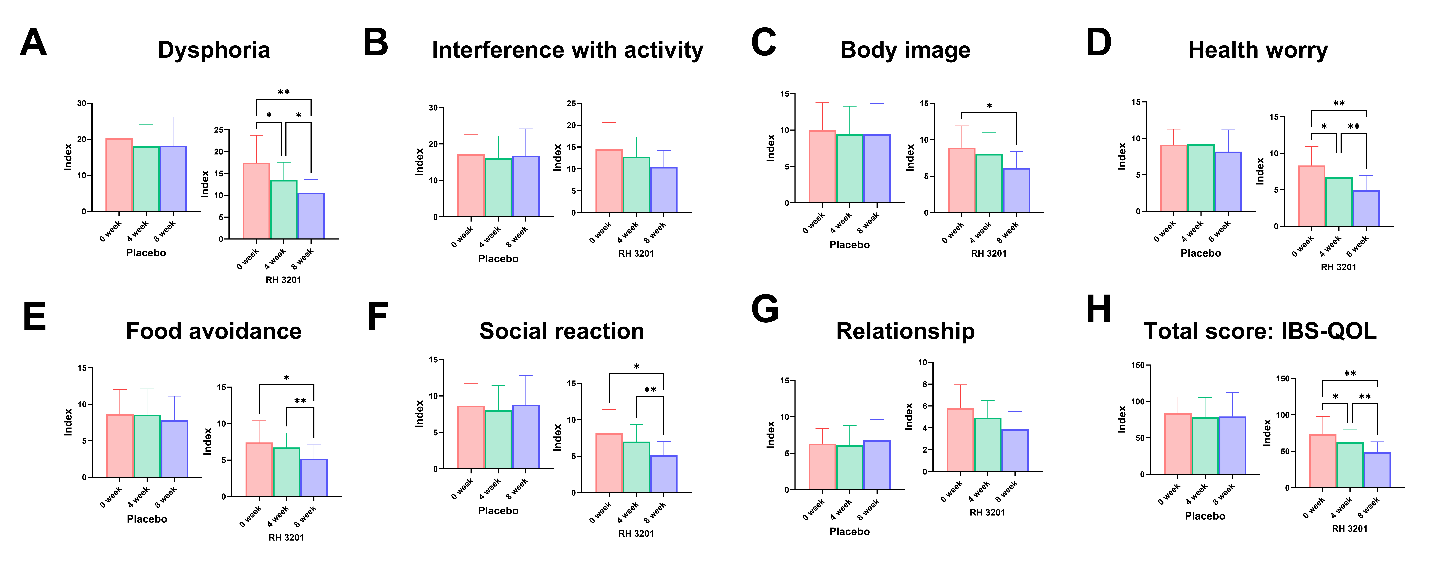


**Supplementary Fig S5. Alpha-diversity indices of fecal microbiome from the placebo and RH 3201 groups.**  (**A**) Chao1, (**B**) Shannon and (**C**) Simpson indices at 0, 4, and 8 weeks of intervention. Significant differences compared to the placebo group are indicated as * (*p* < 0.05) using Mann Whitney U-test.

**
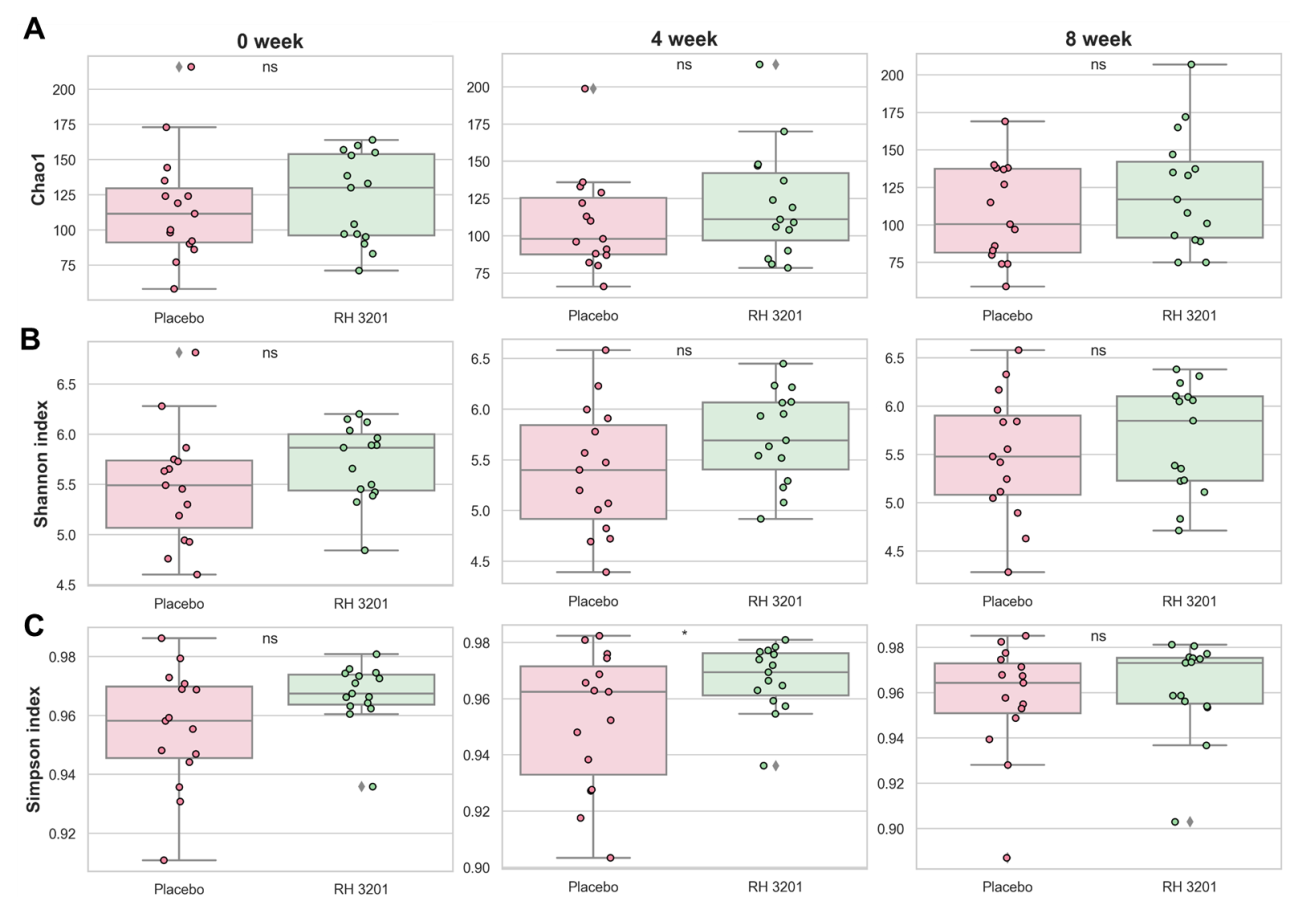
**

**Supplementary Fig S6. Relative abundance of bacterial taxa between the placebo and RH 3201 groups.** (**A**) the phylum level, (B) the genus level, (C) the species level. “Others” are less than 1% of total taxonomic composition.


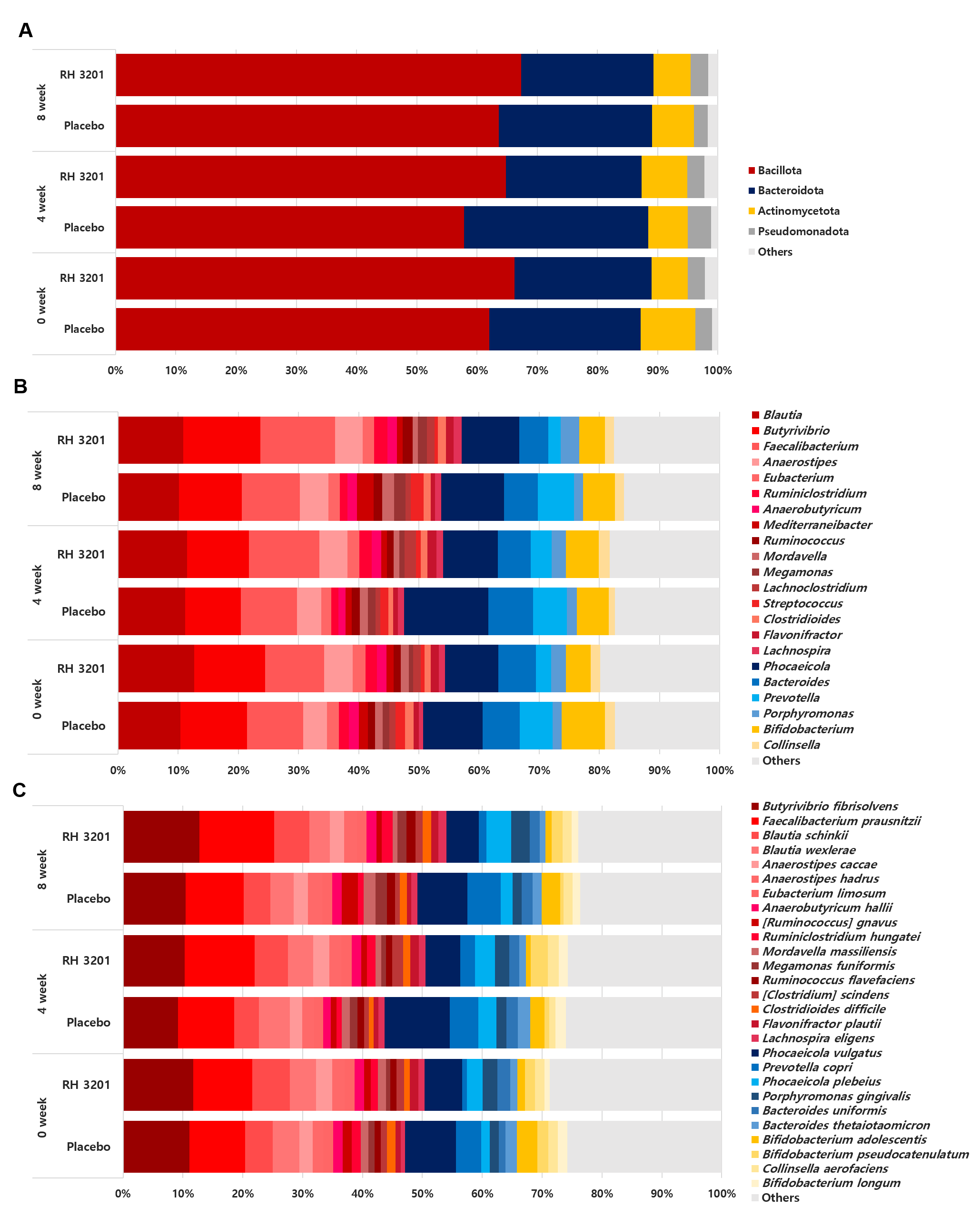


**Supplementary Figure S7.** Metabolite set enrichment analysis (MSEA) of the identified fecal metabolites from RH 3201. The figure shows top 25 enriched metabolic pathways covering the identified metabolites.


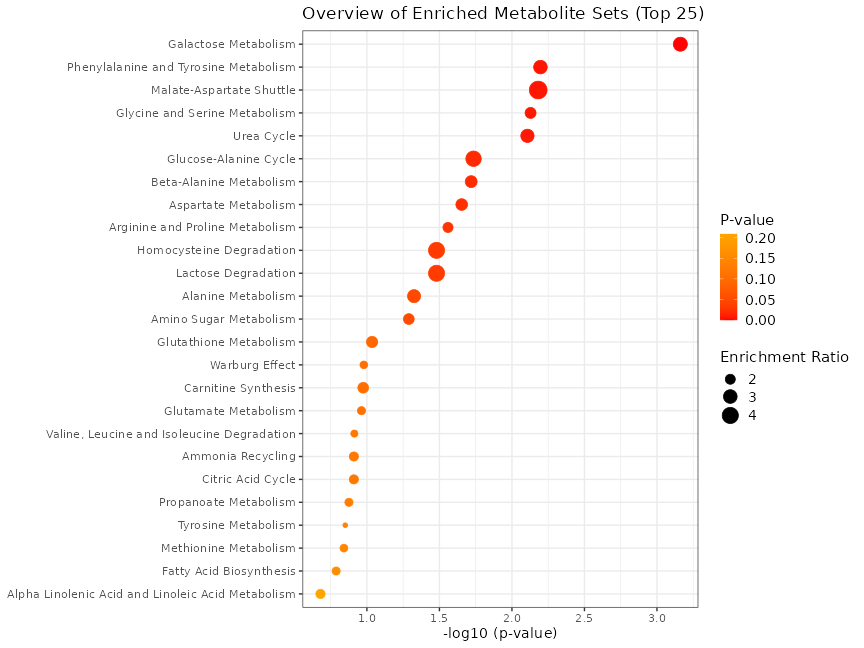


**Supplementary Fig S8**. **Relative abundances of specific metabolites over time of intervention in the placebo and RH 3201 groups.** Metabolites differentially abundant in RH 3201 during the 8-weeks of intervention compared to the placebo group were selected (N-acetylornithine, xanthine, and 3-phenylpropionic acid), and their relative abundance was compared at 0-week (**A**), 4-week (**B**), and 8-week (**C**). Significant differences between the groups are indicated as * (*p* < 0.05) using Mann Whitney U-test.


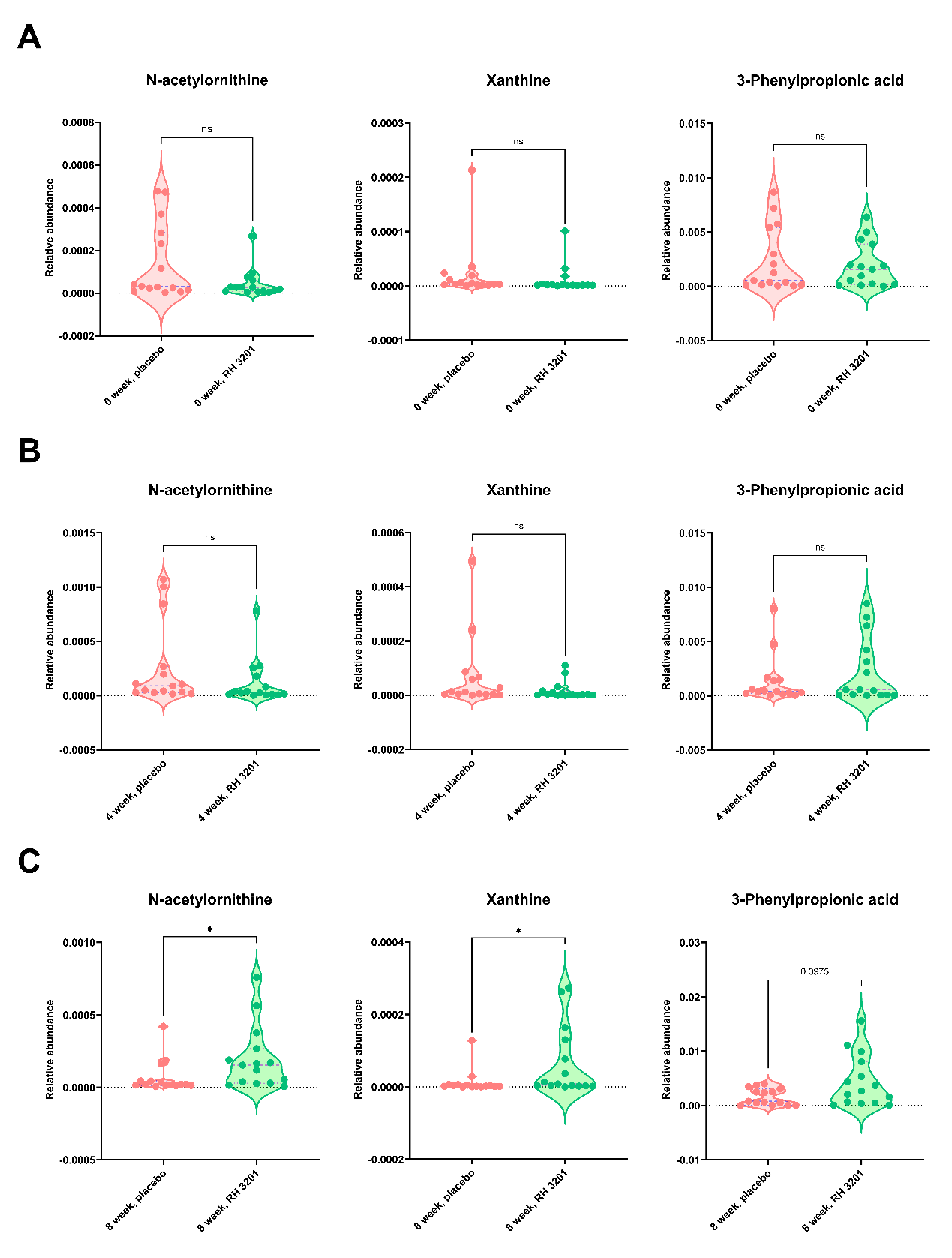


**Supplementary Figure S9.** Spearman correlation analysis of fecal microbiome with N-acetylornithine, xanthine, and 3-phenylpropionic acid. Each row represents whole identified bacterial genera at 8-week, and each column represents the selected metabolites. Red and blue are positive and negative correlation, respectively.


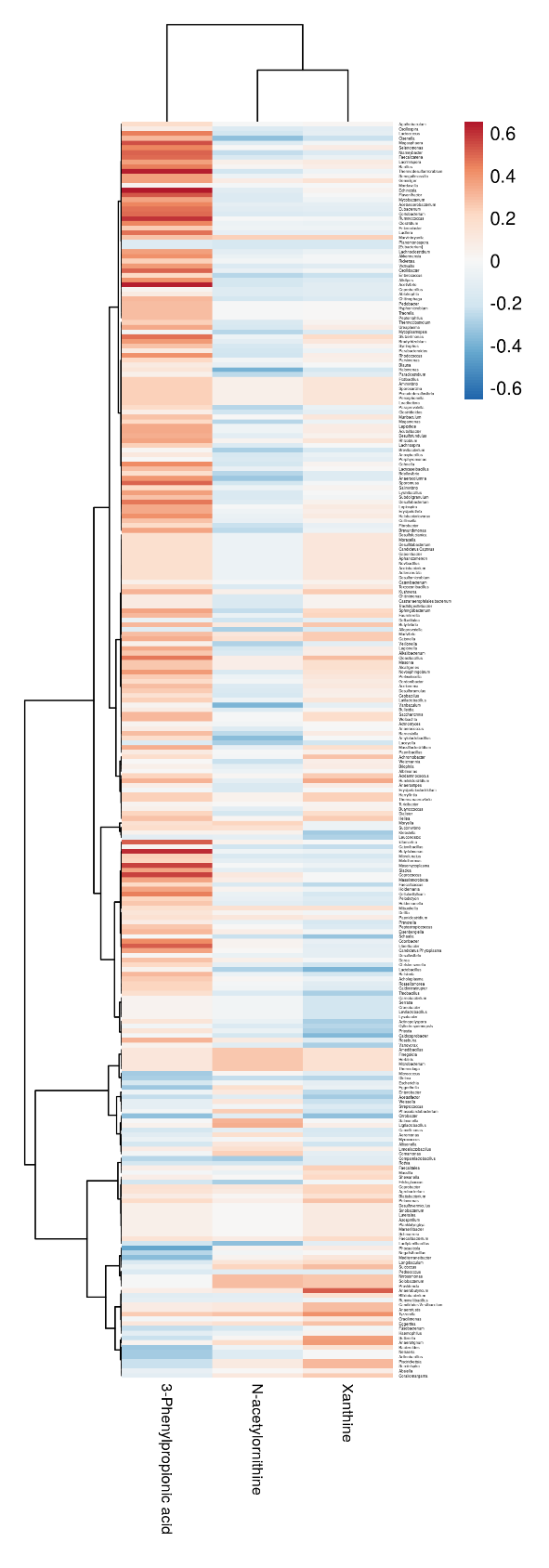

Supplement: Supplementary file 1 — Supplementary Material 1 [file 41598_2024_72887_MOESM1_ESM.docx]
